# Object Recognition, Segmentation, and Classification of Mobile Laser Scanning Point Clouds: A State of the Art Review

**DOI:** 10.3390/s19040810

**Published:** 2019-02-16

**Authors:** Erzhuo Che, Jaehoon Jung, Michael J. Olsen

**Affiliations:** School of Civil and Construction Engineering, Oregon State University, Corvallis, OR 97331, USA; erzhuoche@gmail.com (E.C.); jaehoon.jung@oregonstate.edu (J.J.)

**Keywords:** point cloud, lidar, mobile laser scanning, feature extraction, segmentation, object recognition, classification

## Abstract

Mobile Laser Scanning (MLS) is a versatile remote sensing technology based on Light Detection and Ranging (lidar) technology that has been utilized for a wide range of applications. Several previous reviews focused on applications or characteristics of these systems exist in the literature, however, reviews of the many innovative data processing strategies described in the literature have not been conducted in sufficient depth. To this end, we review and summarize the state of the art for MLS data processing approaches, including feature extraction, segmentation, object recognition, and classification. In this review, we first discuss the impact of the scene type to the development of an MLS data processing method. Then, where appropriate, we describe relevant generalized algorithms for feature extraction and segmentation that are applicable to and implemented in many processing approaches. The methods for object recognition and point cloud classification are further reviewed including both the general concepts as well as technical details. In addition, available benchmark datasets for object recognition and classification are summarized. Further, the current limitations and challenges that a significant portion of point cloud processing techniques face are discussed. This review concludes with our future outlook of the trends and opportunities of MLS data processing algorithms and applications.

## 1. Introduction

Light Detection and Ranging (lidar) technology has revolutionized surveying and mapping through capturing detailed, accurate 3D data to support a plethora of applications. Mobile laser scanning (MLS, also called mobile lidar, ML, or mobile terrestrial laser scanning, MTLS), herein referred to as MLS, functions efficiently from a moving platform throughout the area of interest. Most commonly, the MLS system is mounted to a vehicle and can capture detailed geometric information of the roadway and surrounding area in a form of point clouds. These data are georeferenced by combining the scanner(s) with an inertial navigation system with global navigation satellite system (GNSS) aided to calculate precise, 3D coordinates for each point in the point cloud. For highest accuracy applications, rigorous survey control points are often established or a multi-pass adjustment technique is employed (Nolan, et al. [1,2]). Another attribute of MLS point cloud is intensity, which can be utilized to estimate the reflectivity of the objects after radiometric calibration or intensity normalization (Kashani, et al. [3]). Some MLS systems also integrate camera sensors such that they can simultaneously record a video log and provide color information to the point clouds by assigning the RGB values to each point. 

Given the significant benefits provided by MLS, it is being utilized or adopted by many transportation agencies worldwide (Olsen, et al. [4,5]). MLS datasets can often be used for multiple purposes by multiple persons to support a wide variety of applications, reducing the efforts of field data collection. MLS provides a more thorough field survey compared with traditional surveys with total stations or GPS, minimizing the need for costly repeat visits to the site for data acquisition. The high spatial resolution and accuracy of the MLS data enable derivate products (e.g., DEMs, 3D infrastructure models, etc.) to be completed at an increased level of detail compared with other approaches (Oliveira, et al. [6]). Additional sensors (e.g., inertial profiler to measure pavement roughness, mobile retroreflectometer) can also be integrated onto the mobile platform to capture other information necessary for various applications (e.g., road maintenance) simultaneously with the acquisition of the point cloud data (Olsen, et al. [4]). Lastly, MLS provides safety benefits by minimizing requirements for personnel to work on or adjacent to the roadway. 

A wide range of MLS systems exist. depending on the application(s) of interest and scope of the survey. Puente, et al. [7] described and compared configurations for several MLS systems. Some systems are specialized for certain applications such as pavement analysis whilst others are configured for general 3D data acquisition. Lower cost asset management and mapping systems (typically ~$400 k US) normally achieve sub-meter accuracies at the network (i.e., positioning within a national spatial reference system) level and decimeter accuracies at the local or relative (i.e., measurements within a portion of the point cloud) level. Data from these systems are suitable for locating features of interest (e.g., locations of signs, stoplights, barriers, etc.) for a GIS-based asset management system. However, the data would not be suitable for other applications such as pavement analysis, clearance analysis, or engineering design. Survey grade systems (typically~$1 million US) can achieve centimeter level accuracies at both the network and local level. These systems can provide engineering design quality data with adequate surveying and data processing procedures. 

**Figure 1:**
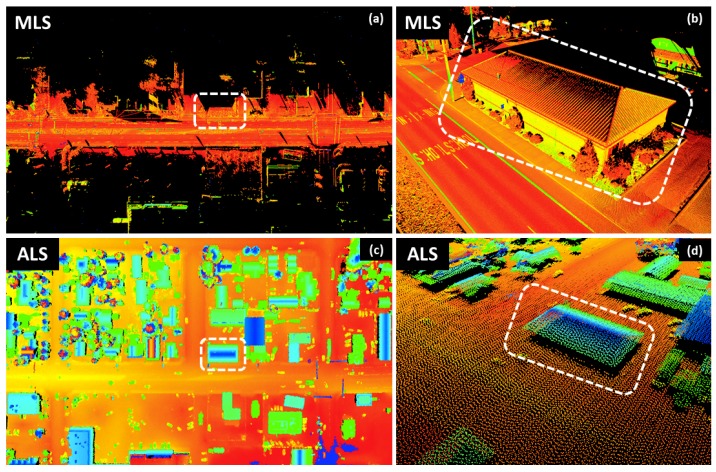
Comparison between MLS (**a**) and ALS (**c**) data for the same area of interest showing a close-up of a building (**b**) for MLS and (**d**) ALS.

Airborne laser scanning (ALS) is the most common form of laser scanning utilized. The majority of data processing algorithms, including feature extraction, segmentation, and classification have been developed for ALS data. Nevertheless, fundamental differences that exist between ALS and MLS limit the straightforward applicability of many algorithms developed for ALS to MLS. These differences are summarized in Olsen, et al. [5]. Herein, we will expand the discussion of these differences to their influence on data processing with an example of MLS and ALS data in the same area (Figure 1): (1) ALS is optimized to look down towards the ground capturing the top of objects whereas MLS looks at objects from the side with a view from the ground. This results in differences in the objects captured. It also affects the ability of the lidar unit to see the ground farther away from the scanner. (2) As a result of the altitude of flight and limited swath width, point density tends to be more uniform in ALS data compared with MLS data. In contrast, MLS systems collect data more densely close to the scanner trajectory with a straight view that degrades with distance from the scanner and increasing angle of incidence. (3) MLS can capture surfaces underneath bridges as well as inside tunnels, but can be quite limited in capturing the top of objects, particularly for tall structures. (4) MLS is generally limited to collect data within a short range (typically 100 m) from navigable roadways, whereas ALS has more flexibility of where data can be collected. (5) For MLS, the GNSS measurements are the major error source while for ALS, the inertial navigation system (INS) and the laser footprint size are the major error sources. The laser footprint is significantly larger for ALS (>0.5m) than MLS (few mm to cm), causing additional horizontal positioning uncertainty with ALS. This results in increased noise in ALS surveys of vertical faces such as building facades. Notice that the data collected by an ALS system at a lower altitude can behave more similar to MLS data; however, it can be challenging to process such data by adopting typical ALS data processing approaches due to the same reasons (Williams, et al. [8]). Further, these low altitudes cannot be flown in urban areas.

Terrestrial Laser Scanning (TLS) is another common form of lidar data acquisition. In general, TLS has a similar scan geometry and range with MLS; hence, some approaches for TLS can be potentially adopted for processing MLS data. Nevertheless, it still should be noted that processing MLS data can face different challenges compared with TLS. First, TLS is more flexible to set up such that several scans surrounding the object can be obtained to ensure that more details can be captured, while MLS has less flexibility due to the accessibility of the platform being mounted to a vehicle. In addition, some MLS systems integrate a TLS scanner on the platform; however, even with the same scanner, the accuracy of MLS data is typically lower than TLS data because TLS acquires scans in a stationary manner while MLS is on a moving platform. Moreover, since a TLS scan can be stored as a structured point cloud, some TLS data processing techniques take advantage of such structure to cope with the variant point density and improve the computation performance (Che and Olsen [9]), whereas MLS data is usually stored in an unstructured format.

There are three primary challenges, in general, when utilizing MLS data for any potential application, as follows. First, an MLS dataset can contain hundreds of millions or even billions of points with geometric, colorimetric, and radiometric attributes, requiring high computer processing resources to handle the large data volume. Second, the point clouds are essentially a set of discrete data records and do not inherently have semantic information. Third, because an MLS system acquires data with a high spatial resolution, noise present in the scene (e.g., moving objects) or unwanted objects are simultaneously captured. 

Fortunately, object recognition and classification serve as important procedures to overcome these challenges for further analysis. Object recognition and classification usually commence with a point cloud segmentation, which groups the discrete points into objects. Such process can enable that the following analysis to classify or recognize an object can be framed to a small subset of the data, which can significantly reduce the computation complexity. Then, after the process of object recognition and classification, the data size can be further reduced by modeling these objects, thereby allowing advanced analyses (e.g., hydrological analysis or visibility analysis) to be conducted with the simplified models rather than the bulkier point clouds. Once the objects are extracted and classified, it becomes more straightforward to remove noise and unwanted objects.

Some recent reviews on MLS have been published. Williams, et al. [8] provide background on the basics of MLS technology and trends, including systems components and software. Then, they focus on applications of MLS through industry projects and academic research, highlighting current challenges and future work to facilitate effective use of MLS by transportation agencies. Puente, et al. [7] compare the performance of select MLS systems available at the time of review and categorize them into mapping and surveying capabilities in terms of accuracy, range, and resolution. Guan, et al. [10] include an in-depth description and discussion for using MLS in road information inventory for detection of road surfaces, on-road objects (e.g., road markings and manholes), and pole-like objects. Gargoum and El-Basyouny [11] review researches utilizing MLS data for extraction of objects on road (i.e., road surface, markings, and edges), roadside (i.e., traffic signs, lamp posts, trees, and utility poles), and for assessment of highways (i.e., road cross sectional slopes, vertical alignment information, pavement condition, sight distance assessment, and vertical clearance assessment). Wang, et al. [12] review various reconstruction algorithms for modeling urban objects (i.e., building rooftop, trees, power lines, roads/bridges, and free-form urban objects), focusing on different types of laser scanning data including ALS, MLS, and terrestrial laser scanning (TLS). Recently, Ma, et al. [13] present a review on the state of the art of road object detection and extraction, which is organized and discussed in three aspects: MLS systems, on-road information extraction, and off-road information extraction.

In this paper, we present a comprehensive review to document the current state of the art in MLS data processing with an emphasis on object recognition and classification. The existing approaches are categorized and summarized with both general concepts as well as technical details included. The limitations and challenges of the state of the art in MLS data processing and applications are further discussed. Notably, our review on the existing methods for object recognition has some similarities and overlap with the extraction of on-road and off-road information (e.g., road surface, road marking, pole-like objects, etc.) presented in the just-released review by Ma, et al. [13]. To clarify the unique contributions of this review beyond that review, several highlights are listed as follows:

- We discuss the impact of scene types (forest, railway, tunnel, and urban/street) on development of MLS data processing methods.
- Object recognition approaches are reviewed with technical details summarized from different perspectives.
- Point cloud classification is also covered and summarized from multiple aspects including segmentation, feature extraction, classification techniques, and achievable classes.
- We summarize available benchmark datasets for evaluating an object recognition or point cloud classification approach.
- General limitations and challenges of the existing approaches for object recognition and point cloud classification are discussed extensively.

The paper is organized as follows: in Section 2, we first provide an overview of MLS data processing and clarify the definitions of terminologies including feature extraction, segmentation, object recognition, and classification in this work. Then, in Section 3, we review common approaches used for feature extraction and segmentation related to object recognition and classification. In Section 4, we discuss how MLS data is collected and processed in different scene types. Section 5 summarizes the approaches for object recognition, which are categorized by the type of objects to extract. Section 6 commences with a discussion on the role of feature extraction in existing classification frameworks and subsequently summarizes existing methods for general classification. Then, Section 7 summarizes available public benchmark datasets for evaluating MLS data classification approaches. Finally, we discuss the challenges in general for all the approaches that have been reviewed in the previous sections and provide an outlook on future research in Section 8 and Section 9, respectively. 

## 2. Overview of MLS Data Processing Workflow

**Figure 2:**
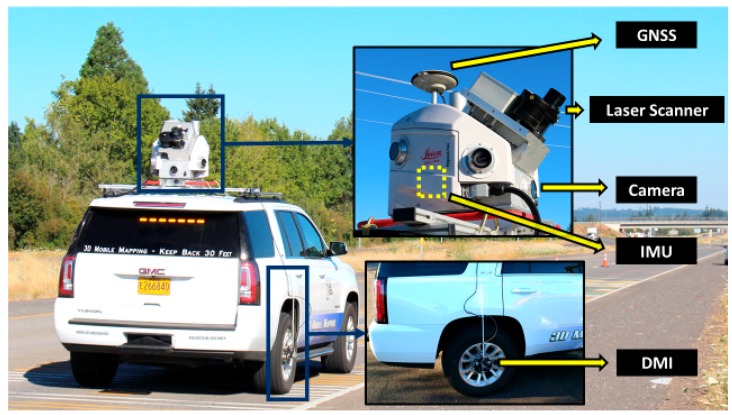
An example of an MLS system (owned by the Oregon DOT) and its components (Leica Pegasus:Two).

During data acquisition with a MLS system, the observations from a GNSS receiver, inertial measurement unit (IMU), laser scanner, distance measuring instrument (DMI), camera, and other sensors are recorded and can be combined based on rigorously determined calibration parameters describing how the sensors are physically integrated (Figure 2). A trajectory recording the time, position, and orientation of the MLS system is usually first obtained using the data from the GNSS receiver, IMU, and DMI. Due to the redundancy in measuring the position and orientation of the system with multiple sensors, post-processing can significantly improve the accuracy and robustness of the trajectory data than a single sensor alone. For example, when the MLS system travels through an area with poor GNSS signals (e.g., under tree canopy, in a tunnel), methods such as Kalman filters can be utilized to predict the trajectory data based on the DMI and IMU observations. This trajectory can further be refined with the addition of ground control or by the multi-pass approach (Nolan et al [1,2]). Once the trajectory has been generated, the geo-referenced or registered point clouds are created in a desired coordinate system (Olsen, et al. [5]). Further processing and analysis are often performed to the geo-referenced point clouds, which will be the subject of this review. 

To minimize confusion and clarify the terminologies we have selected to use in this review, which covers diverse topics from different scenarios, contexts, stages of processing, and research areas, we define four general data processes, as follows: 

*Feature Extraction* is the process whereby a single point or group of points help detect a certain type of point based on low-level attributes. “Low-level attributes” in this case refers to the information without semantics (e.g., location, elevation, geometry, color, intensity, point density, etc.) which can usually be derived from the point cloud data without prior high-level knowledge. For example, planar surface extraction and edge detection are regarded as feature extraction processes in this review.

*Segmentation* refers to the process of grouping points based on the aforementioned low-level attributes into a segment or an object. The segmentation process enables further processing and analysis to be conducted on each segment/object with much richer information compared with processing or analyses considering each point individually in an isolated sense. 

*Object Recognition* is the process to recognize one or multiple types of objects in the point clouds. This process is usually implemented by performing analyses with the results of feature extraction and segmentation with given constraints and rules based on prior knowledge. 

*Classification* is a similar process to object recognition, which assigns a class or identification to each point, segment, or object to represent certain types of objects (e.g., sign, road, marking, or building). The difference between object recognition and point cloud classification is that an object recognition approach is developed to distinguish a few specific objects from others, whereas the goal of classification is usually to label the entire scene semantically. 

## 3. Feature Extraction and Segmentation

Numerous feature extraction and segmentation methods have been applied and developed for processing point clouds. In this section, we mainly focus on the general approaches that are widely used for object recognition and classification, including *Hough Transform*, *Random Sample Consensus (RANSAC)*, *Principal Component Analysis (PCA)*, *Fast Point Feature Histograms (FPFH)*, *Region Growing* and *Connected Components, Graph-Cut, and Supervoxelization.*

The *Hough Transform* presented by Hough [14] was originally developed for detecting lines in 2D imagery. The algorithm includes two steps: (1) transforming the image to the parameter space based on the Hesse normal form of a line, and (2) detecting those lines in the parameter space with a voting procedure. The concept of this approach can be further expanded to detect arbitrary shapes (Ballard [15]) and extended to the extraction of 3D shapes from lidar point cloud data (Vosselman, et al. [16]). The results of a 3D Hough Transform can be directly used to model the scene with pre-defined geometric primitives.

*RANSAC* is another well-known algorithm that can be applied to detect pre-defined geometric primitives (Fischler and Bolles [17]). RANSAC commences with random sampling and determination of inliers and outliers for a target model. Constraints can be applied to the sampling procedure to improve the efficiency. Given the rules (e.g., number of inliers) for selecting the initial model from the previous step, the pre-defined primitives can be detected and further refined. Because RANSAC is generally robust to outliers and noise, there are many methods derived from its basic concept for feature extraction, segmentation, and modeling (Schnabel, et al. [18]).

*PCA* is a data analysis technique that has been widely used for feature extraction from point cloud data (Jolliffe [19]). Different from the Hough Transform and RANSAC with one or multiple pre-defined models as input, PCA is a data-driven process to extract geometric information from an analysis of the local point distribution. Essentially, the results of PCA at a point are the eigenvalues and eigenvectors of the covariance matrix derived by this point and its neighbors. Further analysis can be completed to extract 1D, 2D, and 3D features from the point clouds by metrics derived from the eigenvalues and eigenvectors (Weinmann, et al. [20]).

*FPFH* is proposed by (Rusu, et al. [21]) and has been widely used as a descriptor in various point cloud processing tasks (e.g., classification, registration). For each point, its k-nearest neighbors (kNN) within a given range are searched followed by an analysis of the variation of normals and distance between each pair of points within this neighborhood. Next, the neighbors and point pairs are further optimized to refine the descriptor of the geometric features of a local area. The outcome of Point Feature Histograms (PFH) at a point is a multi-dimensional histogram, which essentially describes and generalizes the local curvature at this point.

*Region Growing* is another data-driven approach applied mostly to segment the lidar data, especially for the complex objects (e.g., Rabbani, et al. [22]). Typically, region growing begins with the selection of seed points followed by an iterative growing process where constraints are provided to investigate the consistency between a seed point and its neighbors. Comparing against the other aforementioned approaches, region growing is more efficient and scalable due to its low computation complexity with less time of traversing through the data given the large data volume (Che and Olsen [9]). 

*Connected Components* is similar to the basic idea of Region Growing and is often utilized for segmenting point cloud data. It is usually performed following a ground filtering process separating the ground and non-ground points, or a point-based classification. Next, by investigating the connection relationship between neighbor points with a set of criteria (e.g., distance), the points lying on the same object can be grouped into a segment. To determine the connected components in a MLS data, kNN or voxelization (Vosselman, et al. [16]) approaches are usually applied. 

*Graph-cut* techniques search for and break the weak connections between nearby points such that the connecting objects within a dataset can be separated. Unlike connected components without further constraints that can under-segment the point cloud as desired in some cases when two objects are partially connected, graph-cut can serve as a refinement approach. There are many ways to implement a graph-cut algorithm such as Min-Cut (Golovinskiy and Funkhouser [23]). Numerous approaches developed for segmenting images can potentially be extended to 3D point cloud.

*Supervoxelization* is also another common segmentation approach for processing point clouds that is expanded from the concept of superpixels in computer vision (Papon, et al. [24]). Supervoxelization over-segments the point cloud by grouping the points into homogeneous segments called supervoxels in terms of various attributes (e.g., normals, colors, intensity, shape, etc.). This supervoxelization can serve as a seeding process for reducing the computation complexity when processing MLS data given its large volume (e.g., Yang, et al. [25]). A supervoxelization usually commences with a normal (regularly spaced) voxelization that groups the points into a 3D grid, and then for each voxel, neighboring points with similar attributes are clustered iteratively such that a supervoxel with an irregular shape is formed. 

**Figure 3:**
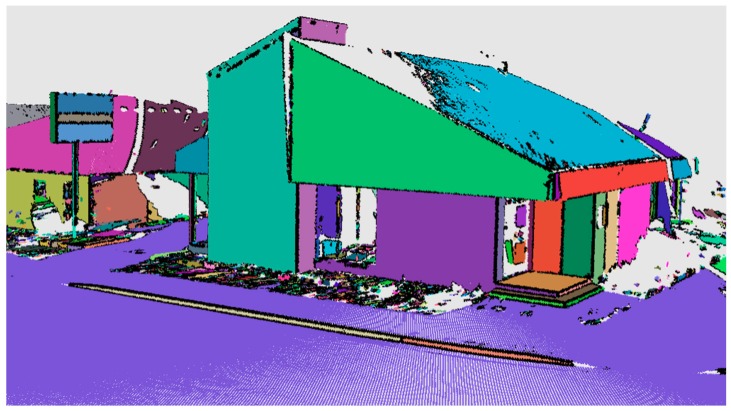
An example of Mo-norvana segmentation results where the black points are the edge points detected while each color represents a segment of smooth surface points.

In addition to the common approaches described above, Mobile Normal Variation Analysis (*Mo-norvana*) is a novel feature extraction and segmentation method proposed by the authors recently (Che and Olsen [26]). This approach detects the edge points first by generating and analyzing a local triangular mesh at each point, followed by a region growing operation to group the smooth surface points (Figure 3). Because the approach reconstructs the scan pattern of how the MLS system acquires data, which can be utilized for generating a grid data structure (scan pattern grid) to organize the point cloud data, *Mo-norvana* is very efficient and can be further sped up by taking advantage of parallel programming (~1 million points per second using eight threads).

## 4. Scene Type

This section covers four types of scenes: forest, railway, tunnel, and urban/street, which encompass the primary scene types for which most algorithms have been developed and tested. The type of scene can significantly influence the quality and reliability of MLS data processing results from a specific algorithm. For example, Meng, et al. [27] analyze several ground filtering algorithms using several ISPRS benchmark ALS datasets representing a range of scene types. The algorithms tested in that review show a wide range of performance in capabilities to handle these scene types.

There are several reasons why algorithms developed for one scene type struggle to achieve reliable results when applied to another scene type. (1) Different assumptions and constraints are built in algorithms based on the objects present in the scene. For example, algorithms developed for urban scenes tend to assume planar structures while algorithms developed for tunnels often assume a cylindrical surface. (2) The strategy for the data acquisition can vary based on the scene type. In the case of a tunnel, the entire tunnel including the walls, roof, and road is generally in view of the scanner, typically at close range. In railway applications, however, the primary objects of interest only occupy a small portion of the view. (3) Noise levels vary significantly between scenes. The algorithms developed for processing will typically apply some noise filtering or outlier rejection criteria that are based on the assumption of the type of noise present in the scene. For example, urban environments contain a substantial amount of well-defined objects such as buildings and structures, but also often contain noise close to the scanner from passing vehicles, bicyclists, pedestrians, etc. In contrast, forested environments do not generally contain well-defined objects but do contain a significant amount of noise from mixed pixels on leaves and vegetation. Additionally, another challenge faced in forested environments is the fact that the objects of interest (e.g., trees) can move during the acquisition as a result of wind or wildlife in the scene. 

### 4.1. Forest

MLS has been utilized for extracting forest metrics along forest roads. Van Leeuwen, et al. [28] provide a comprehensive review on ground-based lidar (e.g., MLS and TLS) and ALS techniques for assessing standing wood and fiber quality. They explain various metrics of interest in forestry applications as well as techniques for extracting these measurements from the scan data. Additionally, Liang, et al. [29] review the advances of using TLS and MLS for the applications of forest inventory. In spite of the high efficiency of MLS in data acquisition, significantly less research has been completed using MLS data than TLS due to the poor GNSS signal and multipath effects in a dense forest. Although most of the work using MLS in a forest require segmentation, feature extraction, and classification for implementation, those papers usually focus on the applications rather than developing automatic algorithms for point cloud processing. A few relevant approaches will be summarized in this section. 

Li, et al. [30] developed a point cloud segmentation algorithm, which first identifies the highest point in a grouping of points as the top of the tree. Their algorithm then implements a growing process to evaluate the points below to determine if the point belongs to the tree based on a spacing threshold rule. Liang and Hyyppä [31] classify ground, stem and crown. Their classification approach evaluates each point and its neighborhood via a *k*-nearest neighbors (*k*-NN) approach. Within the local neighborhood, eigenvectors are computed to define the axis direction and eigenvalues provided the variance of the points along those axes. Tree stems are identified as vertical structures based on the normal vectors. Then, 3D cylinders are utilized to model the individual stem sections. Weighting is implemented to reduce the effects of branches, leaves, etc. Tao, et al. [32] present an approach for segmentation of tree crowns from TLS or MLS data based on ecological theories and principles. They develop a comparative shortest path algorithm that first detects trunks using a density-based spatial clustering of applications with noise (DBSCAN) algorithm given its robustness to noise and efficiency. Then they use the fact that the trunks tend to minimize the distance between the crown and the roots to trace the paths for extracting the crowns. 

These algorithms can also be adapted or extended to other scene types for tree extraction. For example, Herrero-Huerta, et al. [33] develop an approach to extract individual trees in an urban setting to estimate structural parameters such as diameter at breast height (DBH), crown base height, and canopy volume. In their approach, they perform circle fitting via RANSAC to different height bins to determine DBH, voxelization to determine crown base height, and a combination of mesh generation and a-shapes to determine the canopy volume. The position of the tree was determined through PCA. 

### 4.2. Railway

MLS has been used extensively on railway projects whether the system is mounted directly to a rail cart or to a vehicle that is transported on a rail cart. Taking measurement of the railway clearance gauge as an example, Mikrut, et al. [34] analyze and test the performance of both ways to setup MLS systems and conclude that the accuracy for both configurations is sufficient despite of different advantages and disadvantages. Most work focuses on extracting rail tracks and beds (e.g., Blug, et al. [35], Yang and Fang [36], Elberink and Khoshelham [37], Hackel, et al. [38], Stein [39]); however, other techniques (e.g., Arastounia [40], Pastucha [41]) are available for extracting cables, masts, and cantilever supports on the masts. Many techniques implement shape detection/matching or have strict geometric rules to identify the rails and other objects. A key limitation to this assumption is that it can limit the value of the approach for condition assessment when shapes deviate substantially from the template. Table 1 summarizes these approaches. 

For rail tracks, several approaches have been proposed. Blug, et al. [35] evaluate approximately 30 scanline profiles simultaneously in polar coordinates to extract the rail tracks. They also evaluate the angle of the outer rail edge, distance from the scanner to the rails, distance between the two rails, and height differences between head and foot of the rail. Yang and Fang [36] first extract railway beds based on slope within consecutive profiles (Yang, et al. [42]) and then use a combination of geometric attributes (height and slope between head and foot of the rail) and intensity contrast between the ballast and rails to extract the rails. These segments are then connected to amp the railway tracks. Elberink and Khoshelham [37] use local geometric properties such as height and parallelism followed by modeling for fine extraction. The results are smoothed by a Fourier series interpolation. Hackel, et al. [38] extract rail track and turnouts through Support Vector Machine (SVM) (Suykens and Vandewalle [43]) with shape matching. They first identify occluding edges (e.g., depth discontinuities caused by rails) followed by a height evaluation. Then they perform shape matching using iterative closet point (ICP) with a simple, piecewise linear element model. The longitudinal consistency between sections and rail normals are further evaluated for fine-tuning the results. Hackel, et al. [38], [39] uses a 2D (profile) scanner with intensity information to validate the geometric results. They first filter the data to search near the ground and then identify areas with significant changes in distance (i.e., where occlusions occur based on Hackel, et al. [38]) and echo measurements as key points. Objects (e.g. rails) are then identified by template matching.

For extracting catenary systems, Arastounia [40] implements a data-driven approach including that segment and classify the point cloud via an inspection of local neighborhoods with k-d tree. The method initially recognizes track bed evaluating the distribution of height standard deviations to detect the flat area, followed by extracting rail tracks in a similar fashion. Then points above the track bed are recognized to find masts, cantilevers, and the different types of cables using PCA. Pastucha [41] implements a geometric based approach, which searches within a distance of the trajectory and evaluates point densities above the tracks. They utilize RANSAC to detect and classify different objects and then improve the classification with a modified DBSCAN algorithm. 

### 4.3. Tunnel

MLS technology has been explored for maintenance and monitoring of tunnels. The key advantages to MLS over TLS or other techniques include safety and efficiency (the tunnel can remain open to traffic) as well as an improved angle of incidence depending on MLS configuration (Roca-Pardiñas, et al. [44]). The classification techniques are developed for some common applications including deformation monitoring, inspection, clearance, and general asset management/inventory purposes. A recent review (Wang, et al. [45]) summarizes TLS techniques used for tunnel applications, many of which are relevant to MLS. Pejić [46] provides guidance on optimizing scan acquisition strategies. 

Table 2 summarizes the types of objects that can be extracted from tunnels using current algorithms. Most techniques (e.g., Arastounia [47], Puente, et al. [48]) extract a cross section semi-automatically and then fit a model (ellipse, quadric parametric surface modeling) to obtain the measurements of interest or detect specific objects. For example, Arastounia [47] determines the main tunnel axis, extracts cross sections, fits an ellipse to that cross section, and then refines that estimate. These cross sections are then used to extract the side wall, ceiling and floor. Puente, et al. [48] extract lane markings and then evaluate clearances within each lane within the cross section. Puente, et al. [49] apply a height filter combined with an adjusted RGB color histogram to extract luminaries (bright white) from walls (dark surfaces) as the centroid of bright white portions of the dataset. A motion blur correction is applied to improve the extraction results. 

Currently, higher levels of detail in the classification (e.g., ventilation systems, power boxes, etc.) have not yet been addressed; however, such information would be useful to many of the aforementioned applications. Additionally, there is limited work for detailed condition assessment in an automated fashion—rather the assessment is often done manually from the extracted cross sections. Approaches such as Yoon, et al. [50] can detect cracking in concrete lined tunnels; however, detection of smaller cracks (<5 mm) remains elusive with current scanning resolutions. To address this problem, Yoon et al. use a combination of geometric and radiometric data. The radiometric data is analyzed to identify potential installations of steel and plastic, which have lower reflectivity by evaluating histograms for each profile. Then, a distance metric that considers both geometric distance and intensity differences between a point and each of the installation and liner groups is computed to classify the points. Damaged sections are then identified by planar fitting of local segments of the liner surface and evaluating points that deviate significantly from the fitting plane or the plane does not show a normal distribution when compared with the points.

### 4.4. Street

The majority of the literature related to object recognition and classification for MLS data focus on urban and suburban scenes given the prevalence of well-defined, man-made objects throughout these scenes that are well captured with MLS systems. However, the urban and suburban environments provide some substantial challenges for MLS data acquisition. For example, more types of objects (e.g., lampposts, curbs, utilities, buildings, etc.) can be of interest in a street scene while these objects can be rarely seen in other scenes.

## 5. Object Recognition

### 5.1. Ground Objects

Detection of ground objects (e.g., road surface, road boundaries, road markings, etc.) using the MLS data is a challenging task due to: (1) the immense number of points creating a significant computational burden; (2) prevalence of outliers (e.g., passing vehicles, pedestrians, etc.); (3) the lower parts of non-ground objects (e.g., bushes) are often misclassified as ground; and (4) break lines, such as road edges and curbs, can also easily be misclassified as non-ground objects but still are important to include as ground for generation of a DTM (Lin and Zhang [51]). Ordinarily, ground filtering is an essential prerequisite for effective ground object extraction. After filtering the non-ground objects, the ground points can be separated further into road or non-road surfaces by extracting the road boundaries. Finally, road devices (e.g., road markings and manholes) can be identified from the road surface. In general, to extract the ground objects, three ways of organizing the MLS data can be utilized: (1) rasterization, (2) 3D-point, and (3) scanline methods. 

Assuming a planar structure of the road surface over a localized area, the 2D rasterization is commonly adopted for many ground object extraction studies. The key advantage of rasterization is that one can utilize a well-established collection of image processing techniques. Additionally, images can be processed quickly with less memory consumption than 3D point data. Nevertheless, rasterization requires users to prudently select a desired pixel size, which inherently sets the quality of the generated image. A large pixel size can result in loss of details because too many points fall within the same pixel, whereas a small pixel size results in large image dimensions and many pixels with no value, resulting in high computational loads and substantial data gaps, respectively. To avoid these problems in practice, the pixel size is typically selected at a resolution close to the point cloud resolution (Serna and Marcotegui [52]) near the MLS trajectory. To account for the case when several points fall within the same pixel, the following four images are often considered: (1) maximal elevation image, which stores the maximal elevation amongst all points within the same pixel; (2) minimal elevation image, which stores the minimal elevation amongst all points within the same pixel; (3) height difference image, which contains the difference between maximal and minimal elevation images; and, and (4) accumulation image, which stores the number of points within each pixel (Serna and Marcotegui [52]). However, a major drawback to rasterization is that the point cloud resolution can be highly variable with MLS data and degrades rapidly further from the vehicle path. 

Instead of rasterizing the point cloud to generate one or multiple images, 3D-point based methods directly work on the 3D point cloud data. Compared to the rasterization methods, 3D-point methods can preserve more detailed information, but often result in much higher computational loads because of the large data size of point cloud and complexity of 3D geometric calculations compared with 2D operations. To speed up the point querying within an unorganized point cloud, it is ordinarily organized into a data structure (e.g., k-d tree and octree). 

For some MLS systems, the point cloud can be separated into a set of individual scanlines using the GPS time or angle information while other methods simply partition the data into a number of vertical profiles. As a result, the analyses can be conducted in each scanline/profile where a parallel processing can be deployed to further improve the efficiency (e.g., Che and Olsen [53]). The following subsections describe the existing methods for ground, road boundary, and road marking extraction in more detail. 

#### 5.1.1. Ground Extraction

Ground extraction is an essential prerequisite for effective identification of ground objects. Table 3 summarizes existing methods according to three aforementioned categories. In addition, we also summarize the common characteristics (e.g., the point density, elevation variance, and elevation jump) frequently used in the literature for separating ground points and non-ground points in the MLS data.

In the rasterization methods, the λ-flat zone labeling algorithm is widely used to extract the ground (Hernández and Marcotegui [54,55], Serna and Marcotegui [52,56]). Assuming a large planar segment as the ground, the algorithm investigates two neighboring pixels on the elevation image to check if their elevation difference is smaller than or equal to a given λ value for image segmentation, such that the largest segment is identified as the ground. Yang, et al. [57] generate an elevation image from the point cloud using the inverse distance weighting (IDW) interpolation and then apply the discrete discriminant analysis to segment the image into two clusters with the maximum and minimum elevation values. Subsequently, they calculate the optimal threshold maximizing the difference between elevation variances of two clusters, such that the cluster with lower elevation can be identified as the ground. 

In 3D-point based approaches, Husain and Vaishya [58] partition the point cloud into several conjunctive regular square gird cells. For each grid cell, the points below a specified height threshold are evaluated using a sliding circle window; if the standard deviation of z-values of the points within the window is less than a threshold, they are retained as part of the ground. Yadav, et al. [59] also partition the point cloud into a set of 2D square grid cells. For each grid, assuming the Gaussian distribution for the z-values of the ground surface, they iteratively remove the highest points as part of the non-ground objects until the skewness of the distribution becomes zero. Lin and Zhang [51] propose a modified version of the progressive triangulated irregular network (TIN) densification (PTD) method. The original PTD method was introduced by Axelsson [60]: it first divides the entire point cloud into tiles, and then selects the lowest points in each tile as the initial ground points to construct a TIN for the reference surface. The unclassified points in each triangle are evaluated one by one according to the distance to the TIN facet and the angles to the nodes; if a point is found with offsets meeting both an angular and distance criteria, it is classified as a ground point and the algorithm proceeds with the next triangle. In the modified PTD method, rather than working on a single point, the basic processing unit is a segment resulting from the surface growing algorithm, which demonstrates better performance than the classic PTD method. Ibrahim and Lichti [61] propose a density-based filtering to detect the ground points. First, they organize the point cloud through a k-d tree. For each point, its neighboring points within a predefined radius are retrieved for density calculation. In general, the point density is higher along the trajectory of the MLS system and decreases with distance from it. Since the non-ground objects are usually located away from the trajectory, they tend to have a lower point density, and thus they can be distinguished from the ground points. 

In scanline-based approaches, Teo and Yu [62] transform the point cloud into a set of scanlines using the GPS time recorded with each point. For each scanline, if the angle between the normal of a point and the *z*-axis is greater than 5°, it is excluded as a non-ground point. Finally, false-positive ground points returned from the top of cars are removed using an elevation threshold. Wu, et al. [63] propose a vertical profile construction method. It defines a local coordinate system in which the *x*-axis is along the travel direction of the MLS system, the *y*-axis is perpendicular to the *x*-axis in the horizontal plane, and the *z*-axis is upward direction. The points of each scanline are then projected onto the *y*-*z* plane to apply the adaptive α-shape algorithm to identify the candidate ground points. Subsequently, the remaining non-ground points with large elevation variance in the neighborhood are removed. 

Although numerous works on ground filtering have been published, there are several opportunities for improvement in this area. For example, rasterizing the 3D MLS data into 2D or 2.5D images can lead to a loss of information, whereas many 3D point-based methods suffer from high computational expense. In addition, most of the methods in the literature use multiple site-specific parameters to obtain the best result, often requiring a trial-and-error approach to apply a ground-filter successfully to one’s dataset. 

#### 5.1.2. Road Boundary

**Figure 4:**
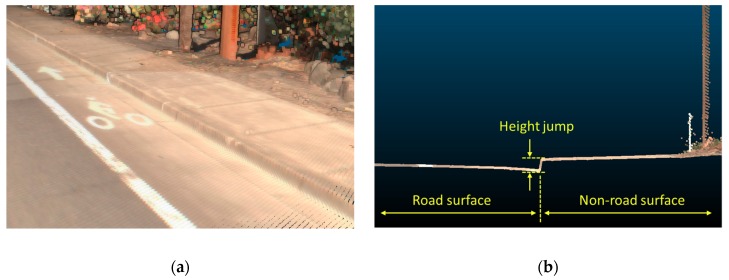
Example of height jump along the road boundary: (**a**) in 3D view and (**b**) in profile view.

Identifying the road boundaries (e.g., curbs, barriers) can help further separate the road surface from the non-road surface (e.g., grass, sidewalk). In the literature, it is commonly assumed that the road surface has a lower height compared to non-road surfaces to extract the road boundaries by detecting the height jump as shown in Figure 4. A list of road boundary extraction methods is summarized in Table 4.

Using a rasterization method, Serna and Marcotegui [56] organize the point cloud into 2D maximal and minimal elevation images, which store only the maximal and minimal heights among all points within each pixel, respectively. Subsequently, they select the minimal elevation image for detection of the curbs because that image usually contains the lowest point on each pixel. For the regions with height changes between 1.5 to 20 cm, the geodesic elongation algorithm is applied to detect the curbs, and then the quadratic Bézier curve strategy is implemented to reconnect the over-segmented curbs. Kumar, et al. [64] rasterize three different datasets (i.e., elevation, reflectance, and pulse width) into images, and then extract the road boundary using the customized snake model. Their approach was shown to be more successful in extracting rural roads compared with other methods, which have been primarily developed for urban roads with a well-defined (e.g., sufficient height or slope difference) road boundary.

In 3D point-based approaches, Ibrahim and Lichti [61] propose a density-based filtering to remove the non-ground objects with a low point density based on the fact that they are usually located away from the system’s trajectory. Afterwards, they detect the curbs by utilizing a derivative of a Gaussian function. Miraliakbari, et al. [65] extract the curbs by investigating the two variants of a jump; one based on height differences and the other based on histograms. A region growing is then conducted for each height criteria to examine the neighborhood. The road boundary candidates are subsequently refined by fitting splines. Xu, et al. [66] propose a 3D voxel gradient analysis that accounts for the number of points in each voxel. An energy function based on eigenvalues is used for thresholding curb extraction from voxels, where the candidate curb edges are connected using a minimum cost path analysis. Yadav, et al. [59] organize 3D points into 2D square grid cells; for each grid cell they iteratively remove the non-road points with the highest intensity until the skewness turns zero based on the assumption of a Gaussian distribution of intensity values,. Then, they compute the local density of neighborhoods for each point, where the points with high intensity variability (e.g., grass-soil surfaces) are excluded as part of non-road surface. Finally, the curbs are traced using the α-shape algorithm and subsequently refined using the B-spline. 

Meanwhile, Zai, et al. [67] propose the use of a supervoxel structure to organize the point cloud into facets. They trace the boundary of each facet using the α-shape algorithm. They set the separation between road boundaries and non-road boundaries as a binary labeling problem and extract the road boundary points using the graph-cut based energy minimization method. In scanline approaches, Miyazaki, et al. [68] transform the point cloud into a set of scanlines using the angle information. They perform a line-based region growing technique to find a set of neighboring line segments to compute the normals to discriminate the curbs from other regions. On the other hand, Cabo, et al. [69] transform the original point cloud into scanlines based on the time information. They use the Douglas-Peucker algorithm (Douglas and Peucker [70]) to simplify the scanlines into a set of straight lines that can be grouped based on the parallelism and proximity between lines. From the end nodes of the road line group, an initial road edge polyline is obtained and then smoothed using a two-stage filtering approach. 

Some methods make use of both rasterization and 3D-point based methods. El-Halawany, et al. [71] compute normals using the PCA algorithm to separate the ground points, which are assumed to have an approximately vertical normal direction. The ground points are then used to generate an elevation image wherein curbs with a sudden height change are detected using the Canny edge detection. Rodríguez-Cuenca, et al. [72] rasterize the 3D point cloud in the elevation image to extract the curb candidates using a binary segmentation. The binary image is then refined using the morphological operation to identify linear-shaped objects as curbs. After which, the 3D curb points are recovered from the binary image to detect the upper and lower curb edges, allowing extraction of curbs occluded or hidden by vehicles. Rodríguez-Cuenca, et al. [73] rasterize the point cloud to produce height difference and accumulation images. A morphological opening operator is used to produce the curb image, from which the 3D curb points are recovered and refined based on the roughness of the point cloud. 

Having reviewed the existing work for road boundary extraction, we found that most of the methods rely on curb extraction. Unfortunately, curbs may not be available for many situations such as a rural area (Zhong, et al. [74]), thereby limiting the use of these algorithms to only extracting well-defined, paved road surfaces in urban areas. 

#### 5.1.3. Road Markings

Road markings are an essential device to navigate and control traffic. Because road markings are made of highly reflective materials (which in turn provide high intensity returns), most studies make use of the intensity information to extract road markings. Table 5 summarizes common approaches found in the literature. 

Road marking extraction often starts with first extracting the road surface. When using MLS data, one can take advantage of the trajectory information to remove the points distant from the scanner location (Guo, et al. [75], Yan, et al. [76]). After this coarse filtering, the road surface can be extracted using the various road boundary extraction techniques discussed above to reduce the area to be analyzed for road marking extraction to the road surface. In addition, it is often desirable to partition the road surface data into smaller subsets to reduce computational complexity (Guan, et al. [77], Yu, et al. [78]). 

Similar to ground extraction, the approaches for road marking extraction can also be categorized into (1) rasterization, (2) scanline, and (3) 3D-point based methods (Table 5). Because the road surface can be considered as a planar structure over a localized area, the majority of the road marking extraction methods in the literature use the rasterization strategy. On the other hand, 3D-point based methods can extract more detailed information, but they often call for high computational complexity. To speed up the process of point retrieval, the point cloud are organized using the data structure such as k-d tree (e.g., Yang, et al. [79], Yang, et al. [80]), octree (e.g., Cheng, et al. [81]), or transformed to a set of scanlines (e.g., Yan, et al. [76], Yang, et al. [82]).

An important aspect of road markings is to represent higher reflectance compared with the road pavement, allowing many approaches to make use of intensity readings for effective marking extraction. However, the intensity values do not directly represent reflectance because they deteriorate with increasing distance, decreased incidence angle, poorer environmental conditions or vary with material or surface properties, resulting in unevenly-distributed intensity values across the same road surface. To obtain uniform intensity values, Cheng, et al. [81] propose a scan-angle based intensity correction model. Meanwhile, a multi-thresholding method, which partitions the road surface to find an optimal threshold for each subset, is adopted in some studies (Yu, et al. [78], Kumar, et al. [83], Guan, et al. [84]).

Intensity-based binary clustering is often used for separation of the road markings from the pavement (Guo, et al. [75], Guan, et al. [77], Yang, et al. [82], Guan, et al. [84], Riveiro, et al. [85], Soilán, et al. [86], Jung, et al. [87]). These road marking segments, however, may include noise which also has higher intensity values compared with the low values obtained on the pavement surface. In the rasterization analysis, to suppress noise while preserving road markings, morphological operations (Guo, et al. [75], Guan, et al. [77], Kumar, et al. [83], Jung, et al. [87]), median filters (Cheng, et al. [81], Riveiro, et al. [85]), and high-pass filters (Cheng, et al. [81]) are found in the literature. Some studies (Yang, et al. [88], Riveiro, et al. [85]) use the Hough transform to extract the linear-shaped road markings from the noise. Meanwhile, Guan, et al. [84] propose a multiscale tensor voting method to filter the noise. Recently in Jung, et al. [87], a statistical approach (i.e., the dip statistics) is implemented to effectively filter out noise with unimodal distribution of intensity values. For 3D-point based analysis, Yu, et al. [78] propose a density-based filtering to discard the points in neighborhoods of lower density as noise. For scanline analysis, Yan, et al. [76] use a dynamic window median filter, which is useful to reduce noise while preserving the road marking edges. After noise filtering, potential road markings can be isolated to produce separate markings using a connected-component labeling process (Guo, et al. [75]) or a region growing technique (Cheng, et al. [81], Guan, et al. [84]). 

With the extracted road markings, some studies are devoted to classify them into different categories using geometric features (e.g., area, perimeter, width, aspect, and orientation). PCA is often adopted to extract such geometric properties by computing the eigen vectors and eigen values (Guo, et al. [75], Yu, et al. [78]). Cheng, et al. [81] propose a hierarchical decision tree using the geometric features to classify the various road markings, such as zebra crossing, dashed and continuous lines, arrows, diamonds, and words. Some other studies use simple template matching where the extracted marking is rotated to the same orientation as the template under comparison, and then the similarity between the marking and a certain template in the database is measured by computing the sum of absolute difference (Guo, et al. [75]) or the cross-correlation (Yang, et al. [82]). Soilán, et al. [86] propose a hierarchical classification using the neural network to separate the arrow and the rectangular markings. The correlation coefficient and Structural Similarity Index (SSIM) method are then performed to classify five types of arrows. More recently, deep-learning neural networks have led to impressive results on the detection and classification of road markings from image data (Li, et al. [89], Tian, et al. [90]). Wen, et al. [91] demonstrate that deep learning can be extended to the MLS data by rasterizing the data into a 2D intensity image. They propose a modified Convolutional Neural Network (CNN) segmentation to detect the road markings, considering both intensity and shape information. Next, the large size road markings (e.g., lane markings and zebra crossings) are identified using the multi-scale clustering based on length and distance between lines. The remaining markings are subsequently classified into dashed lines, texts, arrows, diamond, and triangle markings using a CNN classifier.

**Figure 5:**
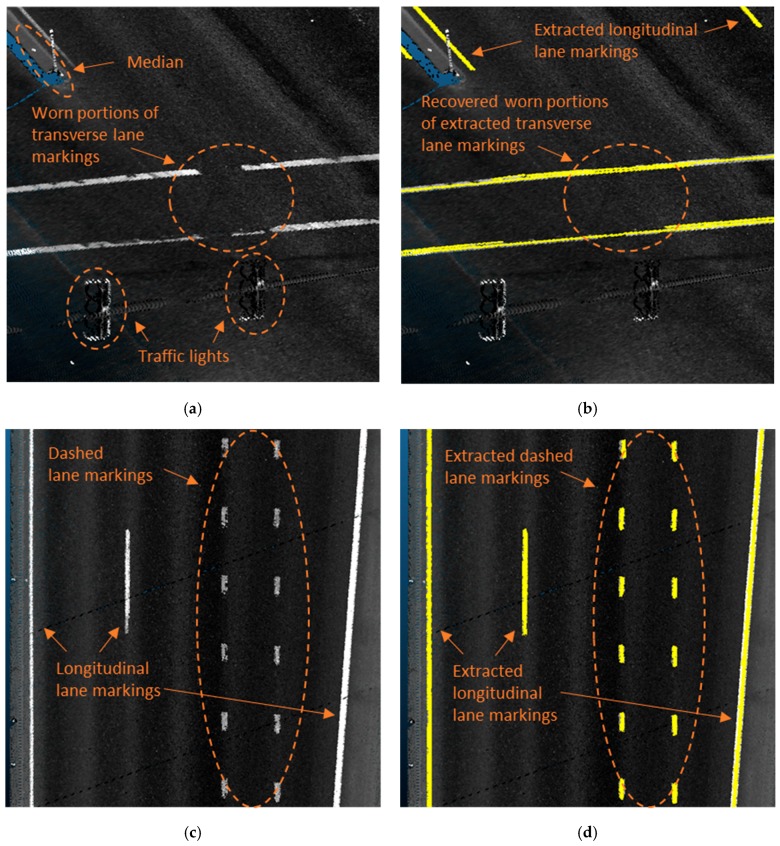
Example of line association: (**a**) worn lane markings (white); (**b**) extracted and associated lane markings (yellow); (**c**) dashed lane markings (white); and (**d**) extracted dashed land markings (yellow).

In summary, rasterization is found to be the most preferred method to extract the road markings because of their planarity (Table 5). Meanwhile, one notable limitation in the literature is that many existing methods are focused on road markings in good condition primarily for the inventorying purpose; in order to extend to which the extracted markings can help agencies assess the marking condition, as proposed by Jung, et al. [87], it is desirable to reconstruct the topologies among the extracted road markings so that the worn portions of markings can be recovered and evaluated as shown in Figure 5a,b. Note that the proposed method evaluate and associate the fragmented lane markings whose end points are less than the minimum distance between dashed lane markings (available in specifications by transportation agencies, e.g., FHWA [92]) such that false association can be avoided as shown in Figure 5c,d. 

#### 5.1.4. Manholes

Manholes built into the pavement are one of the key elements in an urban environment to provide access to conduits for rainwater, waste, steam, natural gas, and other utility networks including telecommunication wires and power cables (Guan, et al. [93]). An automated method of detecting and monitoring manhole covers is therefore of great importance for effective infrastructure management (Yu, et al. [94]). To identify manhole covers, Guan, et al. [93] rasterize the MLS data based on the intensity information. Because the intensity degrades as the scanning distance increases, they partition the intensity image into smaller segments using a pre-defined width. Afterwards, the segments are thresholded separately to identify the manhole covers using a threshold selection method (Otsu [95]), followed by a multi-scale tensor voting to suppress noise. Yu, et al. [96] also rasterize the MLS data into the intensity image. They characterize a disk cover with three parameters (2D center location and radius) and a rectangular cover with five parameters (2D center location, width, height, and orientation) to apply a Bayesian paradigm; this paradigm defines the posterior distribution of the parameter set conditional on the given intensity image and simulates the posterior distribution using a reversible jump Markov Chain Monte Carlo algorithm to detect and model the geometric structure common to manhole covers. In another work by Yu, et al. [94], they use a supervised deep learning model to extract the high-order features from the manhole covers on the training intensity images. The high-order features are then used to train the Random Forest algorithm to identify the manhole covers. 

#### 5.1.5. Pavement

Some studies show the potential use of the MLS data for the maintenance of urban road pavements. For example, Díaz-Vilariño, et al. [97] propose a method to classify the asphalt and the paving stone, focusing on the assumption that the asphalt pavement has lower roughness values compared with the stone pavement. The roughness is defined as the deviation of the normal vectors and four parameters (i.e., arithmetic average, root mean squared, skewness, and kurtosis) derived by roughness are computed for the following analysis. The three case studies are conducted using the MLS data, where the arithmetic average and root mean squared are demonstrated as reliable roughness descriptors. Teo and Yu [62] classify old and new pavements based on normalized intensity values. First, the road MLS points are separated into near- and far-range groups by identifying the peak of a 2nd order polynomial fit to the intensity values. After which, two polynomial functions are used separately for the near- and far-range groups to model the relationship between intensity and scan range. Finally, the Iterative Self-Organizing Data Analysis Technique Algorithm (ISODATA) is carried out for the refined intensity values to classify the old and new pavements.

### 5.2. Pole-Like Objects

Extraction of pole-like objects including traffic signs, street lights, trees, and so forth usually consists of the following procedures: (1) preprocessing; (2) pole-like object detection; and (3) classification of a pole-like object.

#### 5.2.1. Preprocessing

Preprocessing may include various processes such as data structuring, ground filtering, and clustering/segmentation. Data structuring is a common but critical procedure in point cloud data processing. The data structure can help organize the point clouds to enable a variety of basic processes (e.g., nearest-neighbor queries) to be achieved efficiently. For organizing the dataset in a large area, a spatial database is usually generated to improve the efficiency of data query. Additionally, some data structures can simplify the analysis as well as the implementation. For example, voxelization is a common structuring technique used in many pole-like object detection methods by aligning the voxels along *z*-axis. This approach inherently embeds the assumption that a pole-like object is placed vertical. More specific examples of utilizing such data structures in pole-like object detection are shown in the following section. By separating the point cloud into ground and non-ground points, a ground filtering process can reduce the data size significantly when searching for objects that are not part of the ground. Because on-ground objects are usually connected by the ground surface, once the ground points are removed, the objects on the ground can be simply separated via a clustering approach. Pole-like objects can then be readily detected by investigating each cluster. Since segmentation and ground filtering are discussed in Section 3 and Section 5.1.1, respectively, we will mainly focus on detection and classification of the pole-like object in this section, as follows.

#### 5.2.2. Pole-Like Object Detection

To detect the pole-like objects in the MLS data, five characteristics are usually used to define a pole-like object and built into the process and analysis: position, verticality, continuity, shape, and size. We summarize the methods developed for detecting general pole-like objects in Table 6. The discussion of extracting a certain type of pole-like objects will be combined with the discussion on the pole-like object classification in Section 5.2.3. 

• *Position*

Based on the typical location of a pole-like object, several assumptions can serve as constraints during the analysis. The first potential assumption is that a pole-like object is connected to the ground surface, which is usually already extracted in the pre-processing. Some methods utilize such an assumption to either examine if a cluster is on the ground (Li and Elberink [98]), or start tracing objects from the ground surface (Li, et al. [99]). Another assumption that can be used to add more constraints with respect to position is the fact that a pole-like object is usually placed on the side of the road. The road surface and/or building façades can be used to define the inner and outer boundary of the search area along the trajectory. With such boundaries defined via extraction of the road surface and/or the building façades, search area for the pole-like objects can be narrowed significantly. For example, Teo and Chiu [100] detect road lanes such that only the clusters on the side of the road are analyzed. Li, et al. [101] and Rodríguez-Cuenca, et al. [102] extract the building facades to help determine the street width; thus, the pole-like object detection can only take place within such range. 

• *Verticality*

Verticality is an important characteristic to distinguish a pole-like object from other objects on the ground. It can be examined during the ground filtering procedure because ground surface is usually assumed to be approximately horizontal. For instance, Yan, et al. [103] use a threshold of slope during the ground filtering procedure such that only nearly vertical objects remain for further analysis. Similarly, Rodríguez-Cuenca, et al. [102] compute a geometric index to describe the verticality of each point to remove points out of interest. Taking advantage of organizing the point cloud via voxelization, Guan, et al. [104] propose an upward-growing approach to separate the vertical objects and the ground. Such idea of upward-growing can be also implemented during the clustering of the points (voxels) on the ground (Li, et al. [99], Ordóñez, et al. [105]). In addition to checking the verticality locally, Yadav, et al. [106] perform a vertical fit to refine the result of detecting pole-like voxels throughout the scene. Moreover, some methods project the point cloud onto a number of horizontal slices to evaluate the verticality after clustering the point cloud into objects. For example, by examining the deviation (or the displacement of the center points) between adjacent slices, pole-like object candidates can be ensured to be nearly vertical (Li, et al. [101], Yan, et al. [107]). Some other methods add constraints based on the attributes of an entire object from the clustering results. For example, Lehtomäki, et al. [108] compute the main axis of each cluster to obtain the orientation of each pole-like object candidate, while Cabo, et al. [109] calculate the area of an object’s projection on a horizontal plane to detect a pole-like object. 

• *Continuity*

It is critical to consider the continuity when detecting the individual pole-like objects in a scene because MLS data is essentially a set of discrete data points. A process such as segmentation/clustering can reconstruct the connection between each point to its neighbors, which is often included in a framework of pole-like object detection. As clearly shown in Table 6, all the methods listed in this section take continuity into account in detecting pole-like objects where most of them include a clustering procedure. During the clustering, the connection between the discrete points are often reconstructed using the data organized into a specific data structure. For example, if voxelization is used in the framework, the neighbors of a voxel can be defined as its adjacent voxels (e.g., Li, et al. [99], Rodríguez-Cuenca, et al. [102], Ordóñez, et al. [105], Yadav, et al. [106], Cabo, et al. [109], Wang, et al. [110]). Another approach to speed up neighbor searching (e.g., k-nearest neighbor or radius neighbor searching) is to build a tree structure such as k-d tree (e.g., Teo and Chiu [100], Yan, et al. [107], El-Halawany and Lichti [111], Yokoyama, et al. [112]). Then, point clustering based on either a connected component or a growing process can be used to ensure the continuity between the points within an object. In addition to the aforementioned approaches, it is notable that some other methods reconstruct the data into scanlines by using the time stamps associated with each point such that the neighbor points can be derived from the triangular mesh generated between two adjacent scanlines (Fukano and Masuda [113]). Another similar example is that Lehtomäki, et al. [108] segment the point cloud in each scanline first, then merge the analysis results if the segments in two adjacent scanlines have overlap in their horizontal projections. 

• *Shape*

The term “pole-like object” has directly defined the type of objects with respect to its shape. However, in an urban or suburb scene, it is critical to distinguish the building façades and the pole-like objects especially considering they are both off the road, vertical, and captured continuously in the point cloud. Fortunately, a building façade can be usually assumed as a set of planar surfaces while a pole-like object is more like a linear shape such as a cylinder. One simple approach to tackle it is to remove the building façade by detecting large planar surfaces (Li, et al. [114]). Many approaches apply PCA, which has been demonstrated to be an effective approach to describe the linearity of a cluster of points, to detect linear objects (e.g., Teo and Chiu [100], Guan, et al. [104], Yadav, et al. [106], El-Halawany and Lichti [111]). Additionally, because a pole can usually be assumed as a vertical cylinder, a cylinder fitting can be implemented to detect pole-like objects. However, an actual pole-like object is not necessarily a single straight cylinder and may be clustered with its attachments. To cope with such problem, El-Halawany and Lichti [111] consider intensity values during the cylinder fitting to eliminate the noise from flags attached on the poles. Some other methods evaluate the shape of a cluster of points based on the assumption that the horizontal sections along a pole-like object should be circles with the approximately same diameter. To examine the shape of each section, Yadav, et al. [106] remove the false positive detection of pole-like objects by computing the compactness. Some methods compare the differences in diameters of adjacent sections of a pole-like object candidate against a given threshold (Li, et al. [101], Lehtomäki, et al. [108], Fukano and Masuda [113], Li, et al. [115]). 

• *Size*

Size is also a distinguishable characteristic for a pole-like object, which can be straightforward to compute in MLS data. Generally, a pole-like object is designed to be significantly higher than vehicles, pedestrians, bushes, and other objects to ensure its visibility to the drivers and pedestrians. However, despite the fact that in some cases (e.g., an urban scene), the building can often be higher than pole-like objects, only using the height to examine the size of a pole-like object candidate may lack robustness to distinguish a pole-like object from a building. It is generally more straightforward to remove other tall objects such as buildings from the candidates of pole-like objects by computing the width of a horizontal section. Therefore, the height and width of the pole-like object are the two primary attributes used by most methods considering size listed in Table 6. Another noticeable advantage of using the height and width to detect pole-like object is that it is intuitive for a user to select the parameters by approximating the sizes of the target objects because they are often consistent across a scene. 

• *Combination of Metrics*

Some methods combine multiple characteristics into a single metric in order to limit the total number of parameters. For example, Yokoyama, et al. [112] propose a pole-like object detection method using a combination of metrics considering verticality, shape, and size jointly. However, a limitation to this approach is that providing thresholds for such mixed metrics are less intuitive and may require prior knowledge. One possible solution for this is to develop a supervised approach including a training stage that can learn the optimal parameters and weights. For instance, Guan, et al. [104] integrate verticality, continuity, shape, and size into a local descriptor to a feature region and then generate a “contextual visual vocabulary” considering the context between neighbor regions. With such vocabulary generated from the training datasets, the pole-like objects can be detected by matching their representations of “bag-of-contextual-visual-words” to the “vocabulary”. 

#### 5.2.3. Pole-Like Object Classification

**Figure 6:**
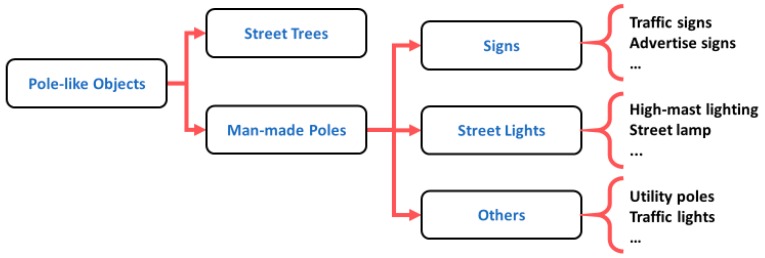
The hierarchical structure used for classifying pole-like objects.

Once the pole-like objects are extracted from the point clouds, they can be further classified into different classes (e.g., trees and man-made poles) based on different rules and methods. Given the fact that the existing methods for classifying pole-like objects are designed to achieve different goals and tested using the datasets with presence of different objects, in this section, we will summarize the approaches and rules that these methods utilize for extracting or classifying a certain type of pole-like objects. The types of pole-like objects are organized into a hierarchical structure for purposes of the following discussion (Figure 6). 

• *Street Trees*

In most cases, the trees only need to be distinguished from the man-made poles (e.g., signs, street lights, etc.). There is a significant difference between a street tree and a man-made pole in terms of geometric shape. However, it is challenging to define the shape of a tree with a one-size-fits-all model to detect all the trees in a dataset due to the irregularities and occlusions caused by branches and leaves. As a result, a measure of point distribution and size are the two mostly used attributes in the existing methods to detect street trees.

In general, the diameter of the tree crown is significantly larger than the tree trunk, such that there are more points lying on the branches and leaves than the trunk; hence, some methods develop a metric of describing the point distribution for a pole-like object. For example, Rutzinger, et al. [116] compute the point density ratio between the number of points on a pole-like object and its lower part because there are significantly more points captured on the crowns than the tree trunk. The trees can be detected by thresholding for the point density ratio and the standard deviation of elevation. Rather than using a vertical point distribution, Zhong, et al. [117] generate a histogram to represent the horizontal point distribution where a tree corresponds to a normal distribution but with a large standard deviation. PCA is another approach to evaluate the local point distribution. Huang, et al. [118] develop a feature vector including the eigen values for each object, and then utilize SVM with a Radial Basis Function (RBF) kernel function to optimize the parameters for distinguishing tree and non-tree objects. Weinmann, et al. [119] extract the features using PCA at each point first and then classify each point using Random Forest followed by clustering the points in the same class. 

In addition to the point distribution, the size of a pole-like object can also be used to recognize a tree. For example, a tree crown usually features a larger radius compared with man-made pole-like objects (e.g., utility poles, street lights). As another example, manmade poles are ordinarily consistently higher or, in some cases, lower than a tree (e.g., a street light or utility pole is usually higher in elevation compared with a tree near the road), so height is utilized for the classification. For instance, Guan, et al. [120] simply apply a threshold of the crown size to filter out non-tree pole-like objects. Wu, et al. [121] analyze the upper and lower part of a pole-like object differently such that it adapts to both tree trunks and crowns. A top-down radius-constrained searching is proposed in the growing process to identify lower part of the tree trunk, followed by a bottom-up neighbor searching to detect the upper part of the tree trunk and tree crown. A potential pitfall of these approaches is that the height assumptions can vary in different urban settings and especially in rural areas where tree heights and species can vary substantially. Similar with different approaches processing the trunk and crown separately, Li, et al. [122] detect the tree crown by computing the radius, roundness, and perimeter of a seed horizontal layer of a pole-like object. In addition to the radius, Rodríguez-Cuenca, et al. [102] compute the mean and standard deviation of roughness to classify the pole-like objects.

Although there are a lot of methods proposed to distinguish trees and man-made pole-like objects, only a few methods (e.g., Guan, et al. [120]) are capable of classifying the trees into different types or species since they are usually not of interest for transportation related applications:

• *Signs*

**Figure 7:**
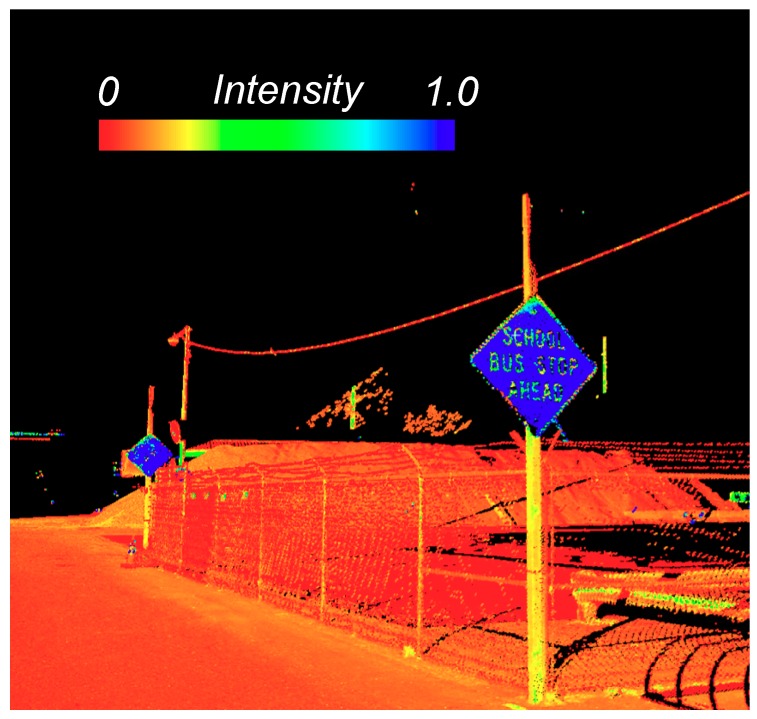
MLS point cloud at a traffic sign colored by intensity values.

Traffic signs are important features in a street scene, and they are vital for the safety of drivers and pedestrians. Thus, an automated approach to extract and classify signs can be helpful for various applications such as asset inventory, sign visibility/occlusion estimation, sign condition assessment, etc. (Wu, et al. [123], Sairam, et al. [124], Huang, et al. [125], Ai and Tsai [126]). Once the pole-like objects are detected, most signs mounted on a pole can be extracted by evaluating planarity. Moreover, traffic signs have a high retroreflectivity for nighttime visibility, which results in a high intensity value (Figure 7). In addition to the requirement of retroreflectivity, traffic signs also follow standards for a traffic sign in terms of color, shape, size, height, and so forth. These characteristics serve as aids in the recognition of different types of traffic signs. The characteristics and machine learning techniques used in existing methods are summarized in Table 7.

Within these characteristics used for detecting traffic signs, to evaluate the planarity of a pole-like object, plane fitting based on RANSAC (Li, et al. [127]) and PCA (Vu, et al. [128], Yang and Dong [129], Riveiro, et al. [130], Soilán, et al. [131]) are the two most widely used approaches. 

For further recognition and classification, a set of rules and constraints can be developed by measuring multiple characteristics such as size, shape, position, and others. To determine the thresholds or train the classifier, supervised machine learning techniques can be utilized (e.g., SVM applied in Yang, et al. [80], Yang and Dong [129], Soilán, et al. [131], Zhou and Deng [132], Wen, et al. [133]). Some other methods (e.g., Yu, et al. [134], Guan, et al. [135]) apply Deep Boltzmann Machine (DBM), which can encode higher level features considering the neighboring variables to help recognize different traffic signs. 

In addition to the methods for MLS data, there are numerous studies in intelligent driver assistance systems based on computer vision (Møgelmose, et al. [136]). Because MLS data often contain a camera log with the calibration information, the computer vision algorithms can be applied to the camera logs with the color information while the point cloud data provides the geometry of an object (e.g., Ai and Tsai [126]). This way, the result of traffic sign recognition and classification can be more accurate and robust.

• *Street Lights*

Street lights/lamps are common road objects that play an important role for the safety of drivers and pedestrians; hence, detecting and modeling street lights/lamps from the MLS data can be useful for generating a road inventory, management, maintenance, and other purposes. Although the street lights are designed and made in a pre-defined shape, height, and size, these attributes of the street lights can vary in different scenes. Thus, a template matching or supervised learning process with certain prior knowledge is usually needed for reliable detection of street lights. 

Luo, et al. [138] propose a Hough Forest Framework to detect light poles where different parts of a street light and their spatial relationship are described for the training stage based on Random Forest. For refinement, the consistent interval between each pair of adjacent street lights is used to filter the false positive detection results. In addition, Wu, et al. [139] extract the lamp from the pole-like object detection results and compute both local and global features followed by a supervised classification based on both SVM and Random Forest. Rather than exploiting machine learning techniques, some other methods match the extracted pole-like object to a template for recognizing a street light based on a descriptor. For example, by modeling the pre-defined head of a street light, Zhang, et al. [140] simply match the point cloud to the model for detecting a certain type of street light. Zai, et al. [141] first automatically segment the point cloud data based on an Euclidean distance clustering followed by refining the segmentation result based on a graph-cut approach. Then in each segment, compute a feature vector at each point including the normals, horizontal distance to the centroid, and normal variance. The similarity of the feature vectors between two objects is examined via the Bhattacharya distance (Bhattacharya [142]). In another study (Zheng, et al. [143]), also using the Bhattacharya distance, analyze the point distribution to distinguish the street light from other objects. Yu, et al. [144] first generate the 3D shape context descriptor to describe the shape of a street light, and then match the extracted pole-like objects to the template to recognize street lights. 

In addition to the street lights, some other pole-like objects can be simply recognized by a set of rules because they are designed for different purposes. For instance, Yan, et al. [103] extract high-mast light, camera pole, street light, train platform light, and parking lot light by giving rules of different heights and the area of their footprints. 

### 5.3. Other Objects

#### 5.3.1. Buildings

MLS data can also be used for 3D building reconstruction, for which most of the existing methods are based on the assumption of near-vertical segments of buildings. To our knowledge, the first work in this area was presented by Manandhar and Shibasaki [145], where they filter ground points using a histogram analysis and then keep only the points with higher scatterness in the z direction for vertical building face extraction. To further remove outliers, they fit straight lines to the points of each scanline, thereby allowing more effective building surface extraction using plane fitting. Rutzinger, et al. [146] and Jochem, et al. [147] first arbitrarily select a set of seed points from the input point cloud. For each seed point, its neighboring points within a certain range are transformed into the Hough space to extract a seed surface. Further points are then added to a seed surface using a surface growing algorithm. Finally, through a vertical plane fitting, only the surfaces having a slope angle of 90 ± 5° from the horizontal plane are retained to represent the building façades. In Wang, et al. [148], a histogram is computed along the z-axis to filter out the highest peak as ground. Subsequently, the remaining points with the normals toward the MLS system are selected to apply RANSAC plane segmentation to extract building façades. Yang, et al. [149] organize the MLS data in the 2D *x*-*y* plane to generate a Georeferenced Feature (GRF) image such that they perform a discrete discriminant analysis to separate the buildings and trees from other objects based on the height information. The eigenvalues are then computed to separate the buildings from the trees based on the differences on spatial distributions of points in 3D space. Finally, RANSAC plane segmentation is conducted to detect the near-vertical building façades. In another study, Hernández and Marcotegui [54] organize the MLS data in the 2D range image to utilize the Hough transform to detect the building façades based on the height information. Likewise, Yang, et al. [57] organize the MLS data into a 2D range image to apply a discrete discriminant analysis to separate the ground and non-ground objects. The non-ground objects are further classified into trees and buildings based on the size and shape estimated from the boundaries of objects. In Cabo, et al. [150], the input point cloud is transformed into separate scanlines. The Douglas-Peucker segmentation (Douglas and Peucker [70]) is used for each scanline to simplify it into a set of straight-line segments. Based on the proximity and parallelism, the line segments are grouped into a plane to extract the vertical surfaces using the least-squares plane fitting. In contrast to other studies, Xia and Wang [151] propose a method to extract independent residential buildings based on the assumption that a building has two dominant directions that can be approximated by a rectangle. Using the region growing method, vertical wall segments are extracted and projected horizontally to detect 2D rectangles as building instances. Afterwards, non-building points are removed using an energy model with local geometric features and planar shape priors. In summary, while existing methods can achieve good results for extracting near-vertical building façades, more efforts should be directed to extract the non-vertical or non-planar structures.

After building façade extraction, some methods are further extended to extract windows and doors by observing opening areas (data gaps). Wang, et al. [148] organize the MLS data into voxels to detect four window edges by investigating the existence of neighbor voxels. Then, windows are localized using the profile histogram, such that the peaks are detected as window edges. However, a prerequisite for this work is to manually exclude the lower levels of buildings because they often have different patterns of openings compared with the upper levels. Arachchige, et al. [152] segment the building façade using a surface-roughness based region growing method. After which, a rule-based partitioning tree is applied to categorize the façade features into walls, windows, doors, and roofs. In another work by Arachchige and Perera [153], they transform the MLS data in the x-z plane to create the 2D TIN using the Delaunay triangulation. The points within the TIN are classified as the boundary points of an opening if the interior angle between two consecutive neighbor points exceeds the maximum angle threshold of 45°. The detected boundary points are clustered using a connected component analysis, and then fitted with geometric primitives such as straight (rectangular shape), curve (arched shape) or bilinear (wedge shape) lines to detect varying shapes of windows and doors. 

#### 5.3.2. Vehicles

MLS data are useful to detect vehicles on the roadside for street parking design and management. In general, the non-ground objects are separated first from the ground, thereby allowing an effective classification of the vehicles from other non-ground objects using a supervised learning technique. Hernández and Marcotegui [55] rasterize the MLS data in the 2D range image and apply the λ-flat zones labeling to generate a ground mask, where the scan holes are detected as non-ground objects. Subsequently, SVM is used to classify the cars, lampposts, and pedestrians. Although some scanner configurations can deal with occlusions to some degree (Lin, et al. [154]), in most cases, only part of a vehicle can be captured in MLS data. To cope with such incompleteness of data, Xiao, et al. [155] propose a model-based approach for complete reconstruction and localization of vehicles. In this work, the MLS data are first classified into ground, building façade, and street objects by using the approach adopted in Hernández and Marcotegui [54,55], Serna and Marcotegui [52,56]. Two supervised learning methods (SVM and Random Forest) are used to classify street objects into vehicles and non-vehicles using the geometric features that describe size and shape at object level and the model features that correspond to parameters of a model fit to an object. The detected vehicles are further categorized into subcompact (mini or small), compact (hatchback), full-size vehicles (sedan, station wagon, SUV, MPV) and vans using the SVM and Random Forest classifications again. One notable limitation associated with model-based approaches is that the training models are generated manually for limited types of objects and thus may not be generic enough.

## 6. Classification

Classification is the process to semantically label each point in the MLS data that can support further modeling and analysis of the scene. There are several methods developed for classification, which can be summarized into three categories based on the input for the classifiers: point-wise classification, segment-wise classification, and object-wise classification. Such way of categorizing the classification approaches is essentially based on the degree of segmentation. A point-wise classification does not include a segmentation procedure, whereas segment-wise and object-wise methods require pre-processing to cluster the point cloud into segments and objects, respectively. Table 8 summarizes the existing classification methods based on the segmentation approach, feature extraction, classification technique, and the classes to be labeled. 

### 6.1. Feature Extraction

Classification requires feature extraction to describe each point, segment, or object during classification. Feature extraction is mainly based on four types of features extracted from the point cloud: geometric, radiometric, colormetric, and contextual features. Geometric features describe a wide range of local attributes (e.g., shape, size, orientation, roughness, normals, density, etc.) at a point, segment, or object. PCA and FPFH often play an important role in extracting such features, which usually contribute the most in a classifier. In addition to the coordinates, many MLS systems also record intensity and color values at each point, which enables radiometric and colormetric features to be extracted. These features can help recognize some objects such as a traffic sign that is painted in a specific color or covered with a reflective material resulting a high intensity value. Furthermore, contextual features take the relationship between neighboring points, segments, or objects into account and usually generate a graph to represent such context. The classification can be more robust with contextual information considered because geometric, radiometric, and colormetric features only describe the point cloud locally. For example, it can be difficult to develop a descriptor based on color and intensity while a geometric descriptor can be complicated computationally. Fortunately, because the cars are usually found on the streets, a contextual feature based on the relative position from an object to the road surface can be effective for distinguishing cars from other objects on the sidewalk (Golovinskiy, et al. [162]). 

### 6.2. Point-Wise Classification

Point-wise classification refers to the approaches that analyze and label each individual point. Munoz, et al. [157] first implement PCA to label each point as linear, surface, or scatter and estimate the normal. Then they model the directional information to the standard Associate Markov Network for further classifying the point cloud. Bremer, et al. [156] present a method to extract geometric features in multiple scales and classify the points with pre-defined rules and given parameters. Nevertheless, more point-wise classification approaches implement a supervised machine learning technique. Weinmann, et al. [20], for example, propose a framework for point-wise classification of point cloud data. In the proposed framework, to compute and extract the local features for each point, a neighbor selection needs to be conducted. Then via feature extraction and feature selection, a supervised classification takes place to label the point clouds semantically. In the framework presented by Weinmann et al., each stage in the framework is tested and discussed with multiple instances using the existing techniques. To improve this framework, Landrieu, et al. [159] perform an initial soft labeling process using a Random Forest classifier. A weighted graph is generated for a smoothing and optimizing the initial labeling results. It is notable that the soft labeling approach also provides confidence/uncertainty of the classification. Point-wise methods are generally demonstrated to be effective and accurate for classification of lidar data; however, the analysis including neighbor searching and feature extraction at each point can be time consuming given the large data volume of the MLS data. To overcome this challenge, Hackel, et al. [158] down-sample the point cloud and build a multi-scale pyramid to help improve the efficiency of neighbor searching. 

### 6.3. Segment-Wise Classification

Instead of analyzing each point with its neighbors in a point-wise classification, a segment-wise classification first segments the point cloud and extracts features from each segment. The classifier can then be trained and label all of the points in each segment based on the features extracted. Although the concepts of point-wise and segment-wise classification are similar, a segmentation can significantly reduce the data volume to improve the efficiency. Moreover, the features extracted from a segment containing several points can be more robust than a single point because statistics can help reduce error. Notice that for a segment-wise classification, each segment is assumed to be homogeneous; hence, most segment-wise classification approaches over segment the point cloud (e.g., voxelization, supervoxelization). For example, Luo, et al. [138] first partition the data into voxels and extract patches from the point cloud. The patches are described by FPFH descriptors, mean RGB values, and height of the patch centroid. Then after the training stage, a patch matching approach is utilized to classify the patches. The results are further refined using graph-cut with a Markov Random Field (MRF) model. Instead of extracting patches via voxelization for over-segmentation, Luo, et al. [160] first segment the MLS data to supervoxels. Then similar to Luo, et al. [138], a graph-based matching and MRF-based refinement are performed for further classification. Instead of using RGB colors, Sun, et al. [161] compute mean intensity values along with PCA-based geometric features at each supervoxel, and apply a high-pass filter to eliminate the local tendency for extracting the contextual features. With all the features combined, Random Forest is finally applied for a supervised classification.

### 6.4. Object-Wise Classification

Some segmentation approaches are able to group the points into objects effectively in the MLS data (e.g., connected component). Once the point clouds are segmented into objects, additional high-level features can be extracted from these objects to help the subsequent semantic labelling process. Such approaches are referred to object-wise classification in this work. 

Similar to point-wise and segment-wise methods, some object-wise classification methods exploit supervised machine learning techniques. For instance, Golovinskiy, et al. [162] first localize the objects based on the height and perform segmentation based on a graph-cut approach. Then the geometric features are extracted in an orientation-invariant manner while the distance from an object to the nearest street and other objects is computed and analyzed as a contextual feature. SVM is finally applied for training and classification for the results of segmentation and feature extraction. With the same feature extraction and classification approach, Lehtomäki, et al. [164] voxelize the point cloud and adopt connected component for segmentation. Aijazi, et al. [163] also organize the point cloud via voxelization and further group the voxels using predefined constraints, followed by classification based on geometric, colormetric, and radiometric features. With more prior knowledge considered during feature extraction, Xiang, et al. [166] first implement a graph-cut based segmentation algorithm and classify the objects using SVM after feature extraction. The features extracted from the segmentation results describe each object in terms of orientation, projection shape and size, point distribution, etc. In addition to the geometric and contextual features, Serna and Marcotegui [52] also exploit the color information from the point cloud to compute the average RGB values for each object. Moreover, Yang, et al. [80] combine point-wise, segment-wise, and object-wise feature extraction and classification, which enables the scene to be interpreted from different levels. While most of the supervised approaches utilize SVM for semantically labeling the objects, some other methods achieve classification of the point cloud by matching the objects to a pre-defined geometric template model, such as Babahajiani, et al. [165].

For a scene with a few object types, once the point cloud are segmented into objects, it is possible and straightforward to implement an unsupervised classification by providing a set of rules and criteria based on prior knowledge. Some of these rule-based approaches can be considered more as object recognition methods, as summarized in Section 5, given the limited types of objects in the scene to classify (e.g., Pu, et al. [137]). Further, based on the prior knowledge of the scene, different objects can be distinguished by using simple rules. For example, Fan, et al. [167] perform a supervoxel-based segmentation and use constraints on normals, height, color, intensity, and shape of an object. Given a more complex scene, Yang, et al. [25] start with segmenting the point clouds into supervoxels and merge them based on their coordinates, geometric attributes, and color values. Then a set of rules are modeled with given thresholds based on prior knowledge for each object in the scene considering geometric and contextual features. Although semantically labeling a scene based on a rule-based classification approach may need many parameters that can be a function of the number of object types, these parameters are intuitive to give and such unsupervised approaches can be implemented without manually training the classifier required by the supervised approaches.

### 6.5. Deep Learning

In recent year, deep learning has become one of the top trending topics in the field of computer vision due to its effectiveness in classification (usually referred to as semantic segmentation in computer vision). Convolutional Neural Networks (CNNs) are the primary architecture that has been used in deep learning methods for classifying/labeling an entire image (Krizhevsky, et al. [168]). Based on CNN, the idea of Fully Convolutional Network (FCN) is proposed and able to tackle the task of segmentation and classification for an image containing multiple objects (e.g., Long, et al. [169]). Such architectures and approaches are also potential in segmenting and classifying point cloud data. However, because most of the deep learning architectures require a regular and structured data as input (e.g., 2D image), the point cloud data needs to be structured by being projected/rasterized to images, or voxelized to 3D grids (Garcia-Garcia, et al. [170]).

In Section 5, we discuss rasterization approaches and review some methods that take advantage CNN to classify the road markings by rasterizing the point cloud. Ma, et al. [13] also has a comprehensive review on recognizing different objects based on deep learning technique. Another way to represent point cloud data in a 2D format is setting a virtual camera for collecting images from the scene. In addition to the RGB color and the depth that can be projected to the image, geometric features (e.g., normals, incidence angels) and radiometric information (e.g., intensity) can also be fed to the deep learning architecture (e.g., Zhuang, et al. [171], Lawin, et al. [172], Qiu, et al. [173]). Instead of converting 3D data to 2D images, some other methods voxelize the point cloud data and develop deep learning techniques that can cope with voxels (e.g., Huang and You [174]). To further improve the robustness of the 3D CNN, other approaches voxelize the point cloud data at multiple scales such that the local features can be extracted and learned adaptively to different scales (e.g., Wang, et al. [175], Roynard, et al. [176]). 

Due to the success of deep learning in semantically interpreting the scene, such ideas have been widely applied and improved in various fields using lidar technique such as autonomous driving and robotics (e.g., Zhou and Tuzel [177]). However, because of different applications, the point cloud data used in autonomous driving and robotics usually contain significantly less points and have much lower accuracy compared with MLS data. Thus, since this review primarily focuses on approaches for processing MLS data, we do not expand the review and discussion to include more deep learning techniques. Nevertheless, these techniques certainly have immense potential to be applied in MLS data processing and future research on this topic is very likely.

## 7. Benchmark Datasets

There are several benchmark MLS datasets that have been manually labeled for training and validating a point cloud classification algorithm: Oakland (Munoz, et al. [178]), Paris-rue-Madame (Serna, et al. [179]), iQmulus (Vallet, et al. [180]), and Paris-Lille-3D (Roynard, et al. [181]). As shown in Table 9, these benchmark datasets are different in terms of lidar systems, the data formats and fields, data size, and classes.

The Oakland datasets are stored in an ASCII format with the coordinates of each point and its semantic labels recorded. However, reading point cloud data in an ASCII format can take significantly more time than a binary format. PLY is a format to store point cloud in both ASCII and binary manners with a header recorded in ASCII format regardless. With such header, although the fields of the data are arbitrary, the users can obtain the necessary information for reading a binary PLY file from the header. There are also other data formats such as ASPRS LAS (Samberg [182]) that are widely used to store MLS data. However, because the fields for each point in earlier versions of the LAS format are pre-defined, LAS is limited in storing some attributes such as scan origin and object ID.

For each data record, the available fields for the data depend on the sensors used on an MLS system. For example, in addition to the intensity values that are recorded by the scanners used on most MLS systems, some scanners (e.g., Riegl LMS-Q120i) can also provide other raw observations such as scan angle, time stamp, number of echoes. The attributes such as the number of echoes can enable some methods to adopt the approaches developed for ALS data to take advantage of multiple returns. With the calibration parameters for the MLS system, the iQmulus dataset also provides the scan origin, GPS time, scan angle, and range for each point, which enables to test the approaches requiring the trajectory data.

The Object ID refers to the result of an object-wise segmentation. Thus, Paris-rue-Madame and iQmulus provide the Object ID as a field for each point to ensure that all of the points with the same object ID have the same class assigned. This can be advantageous for testing the approaches based on an object-wise segmentation because the accuracy assessment can be conducted separately for segmentation and classification. On the other hand, benchmarks without a ground truth for segmentation can only help evaluate a classification approach solely based on the number of points that are labeled correctly compared with those that are labeled incorrectly.

**Figure 8:**
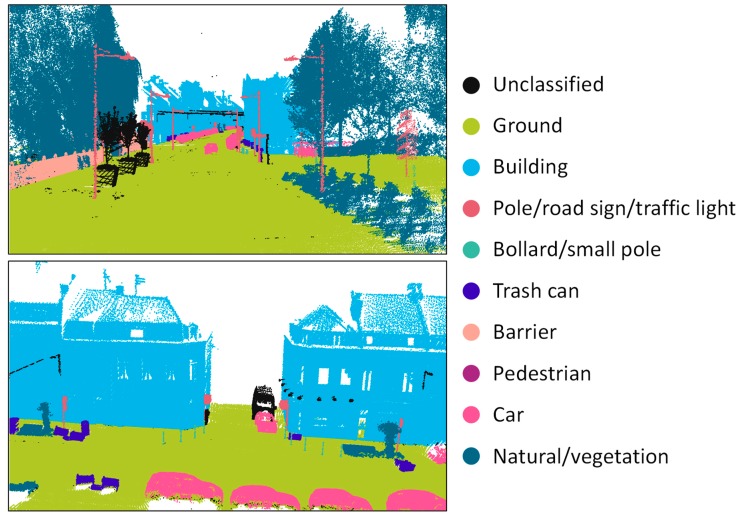
Example of benchmark MLS datasets (Paris-Lille-3D).

Due to different sites and data quality, the classes of the benchmark data are variant. Notice that even though the number of classes are significantly different between the benchmark data, the actual classes with sufficient instances present in the point cloud can be relatively consistent. For example, under the same definition of classes shared by Paris-rue-Madame, iQmulus, and Paris-Lille-3D, the Paris-Lille-3D dataset has 50 classes labeled in the point cloud, among which only 10 coarse classes (including “unclassified”) are recommended because of the limited instances of other classes present in the dataset (Figure 8). 

**Figure 9:**
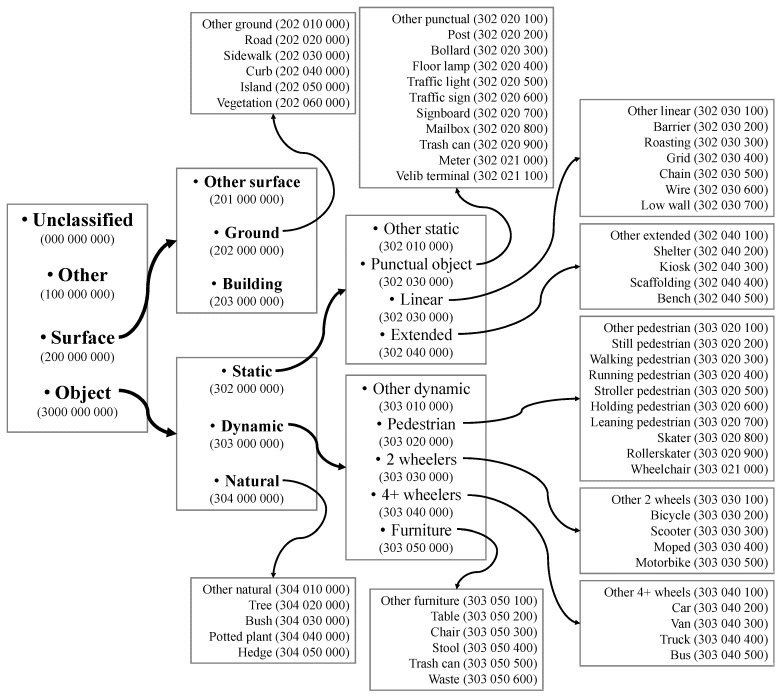
An example of the class tree used in Paris-rue-Madame, iQmulus, and Paris-Lille-3D datasets with the class ID provided.

As shown in Figure 9, a class tree is defined in the Paris-rue-Madame, iQmulus, and Paris-Lille-3D for semantically labeling MLS data. Such a class tree first categorizes the entities into surfaces and objects based on the geometric features. Then the large surface (i.e., ground) can be further categorized into road, sidewalk, and so forth. On the other hand, the non-ground objects are divided into static, dynamic, and natural objects based on if they are man-made as well as their mobility. In most cases, static objects can be considered as road assets; hence, the categorization of static objects is defined considering their representation on a map where linear, punctual, and extended objects should be represented as lines, points, and polygons, respectively. Dynamic objects usually refer to pedestrians and vehicles on the street while natural objects include trees and bushes. Notice the specific classes in the class tree may vary depending on the scene and application.

Other than the class tree, there are also other pre-defined classes. For example, ASPRS defines its standard lidar point classes into 16 classes including unclassified (ASPRS [183]). Nevertheless, such classification is primarily designed for general ALS data, as mentioned in Section 1, which do not typically capture the details (e.g., poles, traffic signs) on a street level due to the scan angle, point density, and data quality. Moreover, unlike the class tree with multiple levels in depth, the ASPRS point classifications do not have a hierarchical structure partially because the classification field in the LAS format only supports up to 256 classes, resulting in limited flexibility in customizing the class coding. It is also worth noting that other standards are developed to help tackle the particular application of utilizing MLS data more effectively. For instance, for asset modeling and management, OpenLSEF [184] not only defines the classes, but also defines the features themselves to be extracted (e.g., center line of the roadway, entrance points of a building, etc.).

## 8. Discussion

Although numerous work has been conducted as discussed and summarized in the previous sections, it is challenging to compare those methods that are tested using different dataset and developed for different applications. A wide variety of metrics are used to assess the quality of the processing. Therefore, in this section, we list several general challenges to be overcome to utilize MLS data in various applications during different stages of data processing for object recognition and point cloud classification. 

• *Impact of Data Acquisition Parameters*

The scanner on an MLS system usually operates at a constant scanning rate and angular resolution; hence, the point density is a function of driving speed, range, and incidence angle. While some methods for data processing and analysis can cope with variant point density, most methods test only data collected in a consistently low driving speed. Faster or variable speed can compromise the level of detail due to the low or inconsistent point density. However, very few works test or discuss the impact of the driving speed during data acquisition. This limitation can be critical because, in practice, the data collection ordinarily occurs at posted speeds, which are much faster than the test datasets. Moreover, MLS systems often only capture those objects within a relatively close range from the trajectory due to occlusions and the degradation of point density with range. Thus, many methods simply remove the points far from the road using a range filter. Nevertheless, the effective range or point density for the proposed methods is rarely tested.

• *Types of Objects*

Most existing methods for object recognition and point cloud classification focus on extracting objects in an urban street scene. However, data collected in suburb and rural highways are rarely tested, which may have different challenges compared with the objects of interest in an urban scene. For example, in some cases, the road is built next to an active rock slope that needs to be monitored periodically such that the rock slope stability can be assessed overtime. It is also vital to ensure the related geotechnical assets (e.g., rock slope barriers, retaining walls) are functional and well maintained. The MLS technique can play an important role for such geohazard monitoring and mitigation as well as geotechnical asset management because of its high accuracy and efficiency in data acquisition. Unfortunately, very few attempts have been made to automatically extract and analyze such natural and man-made objects in MLS data. 

• *Versatility and Robustness*

To interpret the entire scene by classifying the MLS point cloud, all of the classification methods can be simply categorized into unsupervised and supervised approaches. The unsupervised approaches are usually based on a set of rules and criteria where the parameters need to be given based on prior knowledge and fine-tuned through tests. However, the more objects an approach aims to recognize, the more parameters are required, such that the parameter fine-tuning process can be complex. Furthermore, if a hierarchical structure is developed in such unsupervised approaches, the presence or absence of some objects may also affect their robustness. On the other hand, because the supervised approaches are often overfit to the training datasets, processing a new dataset may need additional learning samples or require re-training of the classifier. This process is time consuming since it requires substantial manual labeling. As a result, to demonstrate that a classification approach can be utilized in a practical application, it is challenging, but critical, to quantify the effort required to fine-tune the parameters or manually label the training samples for learning. 

• *Accuracy Assessment*

The effectiveness of most existing point cloud classification methods is evaluated quantitatively via one or more datasets. In most studies, the performance or accuracy of a point cloud classification method is quantified by the number of points or objects classified correctly/incorrectly, particularly for a point-wise or segment-wise classification approach. However, manually labeling the data based on manual or automated segmentation can be subjective, especially in the areas close to the boundaries of an object. In addition, it is still challenging to directly use the result of a point-wise or segment-wise classification due to its discreteness. By contrast, the accuracy of an object-wise classification can be assessed simply by comparing the classification result for each object against the ground truth data. Because the number of objects is much less than the number of points/segments, labeling objects instead of points/segments can be a lot more efficient. Although this way of accuracy assessment is more intuitive to assess the performance of an automatic semantic labeling result, it is still challenging to measure the effectiveness of a classification approach for a particular application such as modeling, asset condition assessment, etc. (Olsen, et al. [5]). Therefore, in some cases, it would be meaningful to assess the performance of a classification method towards its aim or potential applications.

• *Data Volume*

In most of the methods developed for point cloud processing, the computation time follows an exponential growth with increasing data size due to the computation complexity. Some methods report the computation efficiency running on the experimental datasets, which are ordinarily small in size and extent. As a result, it is unclear if the scalability of these methods allows them to be usable to process real-world datasets considering the computation constraints. As summarized in Section 7, only the Paris-Lille-3D dataset contains more than 100 million points while the other benchmark datasets have less than 20 million points, which makes it challenging to test the scalability especially considering the fact that the scan rate of many MLS systems can capture 1 million points per second. Moreover, the large data size also raises concerns in selecting the appropriate data format and the fields to be preserved with the point cloud to achieve the balance between the size of the data file and the information recorded.

## 9. Conclusions and Future Outlook

In recent years, a great number of approaches have been developed for processing MLS data with their accuracy, effectiveness, and efficiency demonstrated. This work reviews the state of the art for semi-automated and automated MLS data processing with an emphasis in object recognition and point cloud classification. The techniques to detect and recognize different objects as well as point cloud classification approaches are reviewed and summarized in terms of both the general ideas and technical details. We also highlight the impact of the scene types and summarize the publicly-available benchmark data. The current limitations and challenges of the existing methods are further discussed. In conclusion, this review can potentially be a guide in improving existing techniques or developing novel approaches for MLS data processing.

In the future, more applications will be able to benefit from utilizing MLS data because the MLS technique is able to acquire detailed spatial data with high accuracy and efficiency. For optimal results, a MLS data processing framework needs to be designed and developed for a specific application. Fortunately, numerous point cloud processing approaches have demonstrated their effectiveness; hence, they can be integrated into a task-oriented data processing framework. Additionally, semi-automatic approaches for processing point cloud data may perform more conveniently and effectively in practice than fully automatic methods given the complexity of the tasks. Thus, developing an efficient framework including both automatic algorithms and user interaction can be considered in some cases. Moreover, data fusion can play a critical role in applications utilizing geospatial data including MLS data. Data collected by different sensors and platforms with different resolution and accuracy capabilities can provide various information and redundancy that support a more accurate, and robust processing, modeling, and analysis. Finally, the research in MLS data processing can also support other state-of-the-art topics and technologies such as autonomous driving, smart cities, sustainable infrastructure, and so forth. 

## Figures and Tables

**Table 1:** Descriptions of object extraction techniques for railways.

Study	Measurement of Interest	Description of Approach	Assumptions/Limitations
Blug, et al. [35]	Rail tracks for clearance measurements for intelligent systems	Scanline profiles in polar coordinates. Evaluates angle of the outer rail edge, distance from the scanner to the rails, distance between the two rails, and height differences between rail foot and rail head.	Straight rails
Yang and Fang [36]	Railway tracks and beds	Slope within consecutive profiles (Yang et al. 2013), height and slope between head and foot of the rail, and intensity contrast between the ballast and rails.	MLS system on the rails. Not suitable to extract railways when MLS acquisition occurs from adjacent railways.
Elberink and Khoshelham [37]	Rail track centerlines	Local geometric properties (height and parallelism) followed by modeling for fine extraction. Smoothed by a Fourier series interpolation	Consistent geometry.
Hackel, et al. [38]	Rail tracks and turnouts	SVM with Shape matching. Identifies occluding edges (e.g., depth discontinuities) followed by height evaluation. Shape matching using ICP with a simple, piecewise linear element model. Fine-tuned by evaluating longitudinal consistency between sections and rail normal.	Consistent geometry-
Stein [39]	Rail tracks and turnouts	2D (profile) scanner with intensity information. Search near ground, identify areas with significant changes in distance, template matching.	Consistent geometry Rails near the common crossing (frog) and blade are too close for detection False positives occur near curbs
Arastounia [40]	Cables (catenary, contact, return current), rail track, mast, cantilever	Data driven approach using k-d tree, the distribution of heights, followed by PCA.	Consistent geometry.
Pastucha [41]	Catenary systems	Geometric based approach, which searches within a distance of the trajectory and evaluates point densities above the tracks. Utilizes RANSAC to classify the points. Projects coordinates to the ground, and improves the classification with a modified DBSCAN algorithm.	Consistent geometry MLS along the railway Potential challenges for extracting railways when MLS acquisition occurs from adjacent railways.

**Table 2:** Descriptions of object extraction techniques for tunnels.

Study	Measurement(s) of Interest	Description of Approach	Assumptions/Limitations
Arastounia [47]	Side wall, ceiling, floor	Extracts cross section along main tunnel axis, fit ellipse, refine and evaluate.	-Only provides measurements at cross sections -Assumes an elliptical tunnel
Puente, et al. [48]	Vertical clearance/cross sections, asphalt, pavement markings.	Generate cross sections and use extracted lane markings to identify lanes for clearance evaluation.	-Only provides measurements at cross sections
Puente, et al. [49]	Road Luminares	Height filter and adjusted RGB color histogram. Apply motion blur correction.	Reliable RGB information is challenging in terms of calibration, image quality, etc. particularly in dark tunnels.
Yoon, et al. [50]	Automated inspection and damage detection (e.g., cracks)	Combination of geometric and radiometric data to identify anomalies.	-Assumes a planar tunnel.

**Table 3:** Summary of studies in ground extraction for MLS data.

Study	Methods			Characteristics		
	Rasterization	3D-Based	Scanline	Point Density	Elevation Variance	Elevation Jump
Yang, et al. [57]	✓	-	-	-	✓	-
Hernández and Marcotegui [54,55], Serna and Marcotegui [52,56]	✓	-	-	-	-	✓
Wu, et al. [63]	✓	-	✓	-	✓	-
Ibrahim and Lichti [61]	-	✓	-	✓	-	-
Husain and Vaishya [58], Yadav, et al. [59]	-	✓	-	-	✓	-
Lin and Zhang [51]	-	✓	-	-	-	✓
Teo and Yu [62]	-	-	✓	-	-	✓

**Table 4:** Summary of method for road boundary detection.

Study	Methods			Characteristics	
	Rasterization	3D-Based	Scanline	Intensity	Geometry
Serna and Marcotegui [56]	✓	-	-	-	✓
Kumar, et al. [64]	✓	-	-	✓	✓
Rodríguez-Cuenca, et al. [72]	✓	✓	-	-	
El-Halawany, et al. [71], Rodríguez-Cuenca, et al. [73]	✓	✓	-	-	✓
Ibrahim and Lichti [61], Miraliakbari, et al. [65], Xu, et al. [66], Zai, et al. [67]	-	✓	-	-	✓
Yadav, et al. [59]	-	✓	-	✓	✓
Miyazaki, et al. [68], Cabo, et al. [69]	-	-	✓	-	✓

**Table 5:** Summary of road marking extraction approaches.

Study	Methods			Characteristics		Classification
	Rasterization	3D-Based	Scanline	Intensity	Geometry	
Guan, et al. [77], Kumar, et al. [83]	✓	-	-	✓	-	-
Guan, et al. [84], Riveiro, et al. [85], Jung, et al. [87], Yang et al. [88]	✓	-	-	✓	✓	-
Guo, et al. [75], Cheng, et al. [81], Soilán, et al. [86], Wen et al. [87]	✓	-	-	✓	✓	✓
Yang, et al. [79]	-	✓	-	✓	✓	-
Yu, et al. [78]	-	✓	-	✓	✓	✓
Yan, et al. [76]	-	-	✓	✓	✓	-
Yang, et al. [82]	-	-	✓	✓	✓	✓

**Table 6:** Summary of characteristics used to define a pole-like object in the existing methods.

References	Position	Verticality	Continuity	Shape	Size
El-Halawany and Lichti [111]	-	-	✓	✓	✓
Fukano and Masuda [113], Wang, et al. [110]	-	-	✓	✓	-
Yokoyama, et al. [112]	-	✓	✓	✓	✓
Ordóñez, et al. [105], Cabo, et al. [109]	-	✓	✓	-	✓
Lehtomäki, et al. [108], Yadav, et al. [106], Guan, et al. [104], Yan, et al. [107], Li, et al. [114]	-	✓	✓	✓	✓
Li and Elberink [98]	✓	-	✓	-	✓
Teo and Chiu [100], Li, et al. [115]	✓	-	✓	✓	✓
Rodríguez-Cuenca et al. [102], Li, et al. [99]	✓	✓	✓	-	✓
Li, et al. [101]	✓	✓	✓	✓	✓

**Table 7:** Characteristics used in traffic sign detection and recognition from MLS data.

Study	Characteristics for Traffic Sign Detection and Recognition						Machine Learning
	Color	Intensity	Planarity	Size	Shape	Others	
Yang and Dong [129]	-	-	√	√	√	-	SVM
Riveiro, et al. [130]	-	√	√	-	√	-	-
Soilán, et al. [131]	√	√	√	-	√	-	SVM
Zhou and Deng [132]	√	√	√	√	-	Position	SVM
Li, et al. [127]	-	-	√	-	√	Height	-
Pu, et al. [137]	√	√	√	√	√	Position	-
Wen, et al. [133]	√	√	-	-	-	-	SVM
Vu, et al. [128]	-	√	-	-	-	Position, orientation	-
Wu, et al. [123]	√	√	√	-	-	Position	-
Yang, et al. [25]	-	-	√	√	-	Height	-
Sairam, et al. [124]	-	√	-	√	-	Height	-
Yang, et al. [80]	-	-	√	√	-	Position	SVM
Yu, et al. [134]	√	√	√	√	√	-	DBM
Guan, et al. [135]	√	√	√	√	√	Height, position	DBM
Huang, et al. [125]	-	√	√	-	-	Orientation, height	-
Fukano and Masuda [113]	-	-	√	-	-	Orientation	Random Forest
Ai and Tsai [126]	√	-	√	-	-	-	-

**Table 8:** Summary of the study on point cloud classification for MLS data.

	Study	Segmentation	Features	Classification	Class
**Point-wise Classification**	Bremer, et al. [156]	-	Geometric	Rule-based	7 (Ground, ground inventory, wall, wall inventory, roof, artificial poles, trees)
	Munoz, et al. [157]	-	Geometric Contexual	Associate Markov Network	5 (Wire, pole/trunk, façade, ground, vegetation); 6 (Façade, ground, cars, motorcycles, traffic signs, pedestrians)
	Weinmann, et al. [20]	-	Geometric	10 classifiers tested	
	Hackel, et al. [158]	-	Geometri Contexual	Random Forest	
	Landrieu, et al. [159]	-	Geometric Contexual	Random Forest	
**Segment-wise Classification**	Luo, et al. [138]	Voxel	Geometric Colormetric Contextual	Graph matching	7 (Road, grass, palm tree, cycas, brushwood, light pole, vehicle)
	Luo, et al. [160]	Supervoxel	Geometric Colormetric Contextual	Conditional Random Field matching	
	Sun, et al. [161]	Supervoxel	Geometric Radiometric Contextual	Random Forest	8 (man-made terrain, natural terrain, high vegetation, low vegetation, building, Hard scape, Scanning artefacts, vehicle)
**Object-wise Classification**	Golovinskiy, et al. [162]	3 approaches tested	Geometric Contextual	4 classifiers tested	10 (short post, car, lamp post, sign, light standard, traffic light, newspaper box, tall post, fire hydrant, trash can)
	Pu, et al. [137]	Connected component	Geometric Colormetric Contextual	Rule-based	3 (Poles, tree, ground)
	Aijazi, et al. [163]	Supervoxel	Geometric Radiometric Colormetric	Rule-based	5 (building, road, pole, car, tree)
	Serna and Marcotegui [52]	Connected component	Geometric Colormetric Contextual	SVM	6 (Car, lamppost, light, post, sign, tree)
	Yang, et al. [25]	Supervoxel	Geometric Colormetric Contextual	Rule-based	7 (Building, utility poles, traffic signs, trees, street lamps, enclosures, cars)
	Lehtomäki, et al. [164]	Connected component	Geometric	SVM	6 (tree, lamp post, traffic pole, car, pedestrian, hoarding)
	Babahajiani, et al. [165]	Supervoxel	Geometric Radiometric	Template matching	4 (Building, road, traffic sign, car)
	Yang, et al. [80]	Region growing	Geometric Contextual	SVM	11 (Ground, Road, Road marking, building, utility pole, traffic sign, tree, street lamp, guardrail, car, powerline)
	Xiang, et al. [166]	Graph-cut	Geometric Contextual	SVM	9 (ground, building, fence, utility pole, tree, electrical wire, street light, curb, car)

**Table 9:** Benchmark datasets for classification of MLS data.

Dataset/Reference	Sensor	Format	Primary Fields	# Points	# Classes	Example Classes
Oakland [178]	Unknown	ASCII	X, Y, Z Class	1.6M	5	Vegetation, wire, utility pole, ground, façade.
Paris-rue-Madame [179]	Velodyne HDL32	PLY (binary)	X, Y, Z Intensity Object ID Class	20.0M	17	Façade, ground, cars, light poles, pedestrians, motorcycles, traffic signs, trashcan, wall light, balcony plant, parking meter, wall sign…
iQmulus [180]	Riegl LMS-Q120i	PLY (binary)	X, Y, Z Intensity GPS time Scan origin # echoes Object ID Class	12.0M	22	Road, sidewalk, curb, building, post, street light, traffic sign, mailbox, trashcan, pedestrian, motorcycle, bicycle, tree, potted plant…
Paris-Lille-3D [181]	Velodyne HDL32	PLY (binary)	X, Y, Z Intensity Class	143.1M	50	Ground, building, pole, small pole, trash can, barrier, pedestrian, car, vegetation…
